# Assessment of Phenolic and Indolic Compounds Removal from Aqueous Media Using Lignocellulose-Derived Surface-Modified Nanoporous Carbon Adsorbents: A Comparative Study

**DOI:** 10.3390/ijms27020804

**Published:** 2026-01-13

**Authors:** Jakpar Jandosov, Dmitriy Chenchik, Alzhan Baimenov, Joaquin Silvestre-Albero, Maria Bernardo, Seitkhan Azat, Yerlan Doszhanov, Aitugan Sabitov, Rosa Busquets, Carol Howell, Sergey Mikhalovsky, Zulkhair Mansurov

**Affiliations:** 1 Institute of Combustion Problems, 172, Bogenbay Batyr St., Almaty 050000, Kazakhstanaitugans@mail.ru (A.S.);; 2 Faculty of Chemistry and Chemical Technology, Al-Farabi Kazakh National University, 71, Al-Farabi Avenue, Almaty 050012, Kazakhstan; 3 Laboratory of Engineering Profile, Satbayev University, 122/22, Baitursynov St., Almaty 050012, Kazakhstan; 4 Laboratory of EPR spectroscopy, Institute of Physics and Technology, 11, Ibragimov St., Almaty 050000, Kazakhstan; 5 Laboratorio de Materiales Avanzados, Departamento de Química Inorgánica, Universidad de Alicante, 03690 Alicante, Spain; 6 Departamento de Química (DQ), Faculdade de Ciências e Tecnologia (FCT), Universidade Nova de Lisboa (UNL), 2829-516 Caparica, Portugal; maria.b@fct.unl.pt; 7 School of Life Sciences, Pharmacy and Chemistry, Kingston University, Penrhyn Road, Kingston upon Thames KT1 2EE, UK; 8 Enteromed Ltd., 85 Great Portland St., London W1W 7LT, UK; 9 ANAMAD Ltd., Sussex Innovation Centre, Science Park Square, Falmer, Brighton BN1 9SB, UK; sergeymikhalovsky@gmail.com; 10 Chuiko Institute of Surface Chemistry, 17, General Naumov St., 03164 Kyiv, Ukraine

**Keywords:** nanoporous carbon adsorbents, physicochemical activation, uremic toxin precursors, adsorption mechanism

## Abstract

P-cresol, indole and indole-3-acetic acid (IAA) are catabolites of amino acids, formed by the gut microbiome. Most of these aromatic hydrocarbon derivatives are excreted by the colon before reentering the body to form “exogenous” protein-bound uremic toxins (PBUTs), which aggravate chronic kidney disease (CKD). Removal efficiencies of these PBUT precursors from model phosphate-buffered saline solutions by three different surface-modified nanoporous carbon adsorbents (PCs) were studied. PCs were produced by physicochemical and/or acid base activation of carbonized rice husk waste. Removal rates achieved values of 32–96% within a 3 h contact time. High micro/mesoporosity and surface chemistry of the N- and P-doped biochars were established by N_2_ adsorption studies, SEM/EDS analysis, XPS and FT-IR-spectroscopy. The ammoxidized PC-N1 had the highest adsorption capacity (1.97 mmol/g for IAA, 2.43 mmol/g for p-cresol and 2.42 mmol/g for indole), followed by “urea-nitrified” PC-N2, whilst the phosphorylated PC-P demonstrated the lowest adsorption capacity for these solutes. These results do not correlate with the total pore volume values for PC-N2 (0.91 cm^3^/g) < PC-P (1.56 cm^3^/g) < PC-N1 (1.84 cm^3^/g), suggesting that other parameters such as the micropore volume (PC-N1 > PC-N2 > PC-P) and the interaction of surface chemical functional groups with the solutes play key roles in the adsorption mechanism. N-doped PC-N1 and PC-N2 have basic functional groups with higher affinity with acidic IAA and p-cresol. The ion-exchange mechanism of phenolic and indolic compound chemisorption by nanoporous carbon adsorbents, modified with surface N- and P-containing functional groups, has been proposed.

## 1. Introduction

P-cresol (para-cresol, 4 methylphenol) and indole are precursors of major uremic toxins, such as p-cresyl sulfate (PCS) and indoxyl 3-sulfate (IS), and, in the case of renal disease, are related to oxidative stress caused by reactive oxygen species (ROS) and cardiovascular disease (CVD) [1,2,3,4,5,6].

Together with indole-3-acetic acid (IAA), they are toxic catabolites that are generated from the fermentation of amino acids, e.g., phenylalanine and L-tyrosine for p-cresol and tryptophan for indole and IAA by the proteolytic community of intestinal bacteria, such as *Bacteroides*, *Bifidobacterium*, *Lactobacillus*, *Clostridium* and *Enterobacteriaceae* families, i.e., *E. coli* spp., etc. Specifically, hydroxyphenylacetate decarboxylase catalyzes the formation of p-cresol from phenylalanine and L-tyrosine, whilst tryptophanase and tryptophan 2-monooxygenase catalyze the formation of indolic compounds, such as indole and IAA, from tryptophan by the intestinal microbiome, respectively [1,5,7].

In the case of healthy individuals, with well-balanced gut microflora and the absence of chronic kidney disease (CKD), these substances are mainly excreted with feces; p-cresol undergoes sulfation by sulfotransferases of colon microflora to form PCS, which can be absorbed through the gut epithelial barrier to enter the bloodstream. Minor amounts of p-cresol and indole can also translocate to the liver from the intestines through a portal vein, where, in hepatocyte microsomes, they undergo sulfation by sulfotransferases to form PCS and IS [2]. Specifically, indole is hydroxylated by the cytochrome P450 enzyme to form 3-hydroxyindole (indoxyl) first, which subsequently is sulfated by the SULT1A1 enzyme to form IS [5,8,9].

Both PCS and IS, as well as IAA, have strong affinity for plasma proteins and form conjugates called protein-bound uremic toxins (PBUTs); the latter undergo renal clearance in the renal tubular cells, causing only minor cumulative damage to the kidney [6,9,10]. Over 10 percent of the human population sustains intestinal barrier dysfunction (IBD), or so-called “leaky gut”, on top of CKD advancement, and this means higher intestinal barrier permeability and absorption of toxins, including uremic toxins and their precursors, which can expedite CKD to end-stage renal disease (ESRD). Moreover, protein-bound IAA, identified as a PBUT by recent studies, can directly aggravate CVD progression, enhancing established risk factors such as diabetes and hypertension, causing systemic inflammatory response syndrome (SIRS), intensifying oxidative stress and triggering vascular calcification, ultimately enhancing risks of cardiovascular morbidity and mortality in CKD patients [2,6,11]. P-cresol, as one of the most widespread phenolic compounds, is a common environmental contaminant of soil and water bodies. Anthropogenic sources of phenols such as oil refineries and pharmaceutical and polymer industries are the main cause of water contamination. Due to its high toxicity, p-cresol is harmful even in low concentrations and is classified by the US Environmental Protection Agency (EPA) as a possible carcinogen [12,13].

Indole has been identified as an essential, versatile signaling molecule with broad environmental distributions and wide spectrum of biological activities; it is extensively used in the pharmaceutical industries [14]. On the other hand, indole is a pollutant in industrial and agricultural wastewater. A relatively high concentration of indole has been found in coal tar and in animal feces and sewage. Studies have shown that indole can cause hemolysis, hemoglobinuric nephrosis, tumor formation and other animal and human pathologies [15].

IAA is the most abundant and the key generic natural plant hormone of the auxin class essential to the world food supply. However, overuse of plant growth regulators leads to soil and thereby groundwater contamination, and via the food chain it can cause detrimental accumulated toxic effects on the environment affecting living creatures, including animals and human beings [16].

Humans are exposed to these uremic toxins via two separate pathways, termed intrinsic and extrinsic, through endogenous or exogenous sources, respectively [6]. Most of these molecules exert biological functions through receptor-mediated signal transduction pathways, including the aryl hydrocarbon receptor (AhR) and Toll-like receptor-4 (TLR4), xenobiotic sensors, expressed in various cell types within the gut. These indoles can have immunomodulatory effects and are potent agonists for AHRs, which regulate host immunity and barrier function at mucosal sites [6,14,17]. In the literature, the terms “endotoxin” and bacterial lipopolysaccharide (LPS), which plays a key role in pathogenesis and endotoxemia and stimulates the innate immune system via triggering the TLR4, are often used as synonyms. For this reason, the classification of LPS (or endotoxin) as an exogenous hormone rather than as a toxin in a strict sense has been discussed [14,18]. Hence, there is dichotomy in the terminology, i.e., whether to consider indole and its derivative IAA as intrinsic exogenous PBUT precursors or as xenobiotics, exogenous phytohormones of the auxin class [19,20].

Adsorptive therapies such as enterosorption, or administration of oral adsorbents, represent an innovative strategy for addressing uremic toxin removal in CKD patients [6]. The synthetic polymer-derived highly porous carbon enterosorbent AST-120 (KREMEZIN^®^, Kureha Inc., Tokyo, Japan), with a large surface area over 1600 m^2^/g, was proven in clinical trials to lower clinically relevant uremic toxins plasma concentrations and ameliorate oxidative stress caused by reactive oxygen species (ROS) in CKD patients [2,4,5,8]. Coconut-shell-derived NaOH-activated PC and apricot-stone-derived H_3_PO_4_-activated PC were used in p-cresol (PCS precursor) adsorption studies, demonstrating good adsorption capacity [13,21].

As a natural siliceous lignocellulosic precursor, rice husk (RH) is an abundant agricultural by-product with minimal commercial value. It is traditionally burned or disposed of in landfills in rice-growing countries, however, RH incineration leads to air pollution and is ineffective due to its low calorific value and high ash silica content which makes RH poorly biodegradable [18]. Converting RH into PC with good adsorption properties not only could add substantial value to the resulting composites, such as carbon/silica adsorbents and catalysts [22,23], but also reduce the inherent problems associated with the current methods of this biomass waste disposal, offering a “greener technology” approach. Recently, there has been a renewed interest in utilizing RH-based PCs as a low-cost sorbent for removing organic pollutants and heavy metals from aqueous media [18,24]. In terms of potential advantages, it is a renewable bioavailable material with a low content of potentially toxic substances, which is important for biomedical applications. The nanoscale silica phytoliths contained in RH serve as a template to create additional meso/macropore space in PC, within the nanoscale range [25,26]. In our previous study, we demonstrated that RH-derived/KOH-activated/ammoxidized (PC-N1) and RH-derived/H_3_PO_4_-phosphorylated (PC-P) enterosorbents had a large surface area and large micro/mesopore volumes. PCS and IS adsorption profiles for PC-N1 and PC-P exhibited good dynamics within 60 min and the sorption degree values lay within 75–99%. Biocompatibility of PC-N1 and PC-P assessed in vitro with Madin–Darby canine kidney (MDCK) epithelial cells showed no evidence of cytotoxicity; the cell survival rates exceeded that of the commercial enterosorbent “Adsorbix Extra” (Norit Ltd., Glasgow, UK). Efficiency of PC-P enterosorbent was also assessed in vivo using a CKD animal model; its administration reduced both the uremia (urea and creatinine levels) and endogenous intoxication [26].

Since p-cresol, indole and indole-3-acetic acid are produced in the gastrointestinal tract (GIT) as catabolites of amino acids before entering the portal circulation and liver and undergoing transformation into PBUTs, it could be therapeutically advantageous to capture them in situ in the colon using enterosorbents. The study results suggested that the obtained PCs are non-toxic, biocompatible and may be used as oral adsorbents, as well as components of water filters, embedded into a macroporous cryogel matrix, for removal of polar micropollutants from water [27,28,29].

The goal of this work is to examine the efficiency of rice-husk-derived porous carbons to adsorb p-cresol, indole and indoxyl-3-acetic acid that can be considered as either aromatic hydrocarbon xenobiotics or endogenous precursors of uremic toxins with a view of exploring their potential as enterosorbents and adsorbents for water treatment. The role of the surface chemical functional groups in the adsorption mechanism of these compounds is also a subject of the proposed work.

## 2. Results and Discussion

### 2.1. Structural Morphology of Surface-Modified PCs

#### 2.1.1. Low-Temperature Nitrogen Adsorption of Nanoporous Carbons

**Figure 1:**
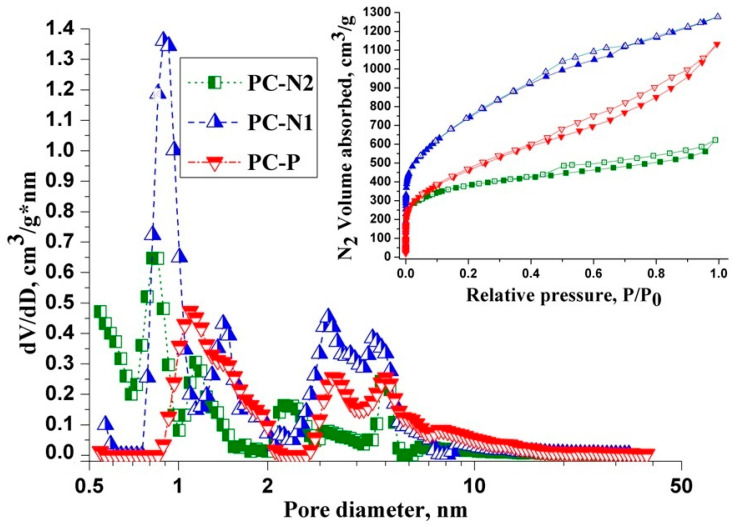
QSDFT–pore size distribution curves derived from nitrogen adsorption–desorption isotherms data for the PC samples.

The pore size distribution (PSD) of PC samples was calculated from low-temperature nitrogen adsorption–desorption isotherms using the slit-cylindrical quenched solid density functional theory (QSDFT) equilibrium model are shown in Figure 1.

According to the IUPAC classification [30], the N_2_ adsorption–desorption isotherms of all the PC samples are a mix of type I (at the beginning of the isotherm) and type IV (at the end of the isotherm). However, a notable steep slope at low relative pressure (P/P_0_ ≤ 0.1) presented in the type I (a) isotherm for the PC-N1 sample indicates abundant narrow micropores (width < 1 nm), while PC-N2 and PC-P samples, presented in the I (b) type isotherm, were associated with a broader range of pore size distributions (Figure 1), including wider micropores and possibly narrow mesopores (<2.5 nm). Each isotherm, a mix of H3 and H4 types, also shows a distinct hysteresis loop at intermediate to high relative pressures, which is characteristic of the presence of slit-shaped large micro- and small mesopores. This is clearly shown on the graphs of the PSD in PC samples, which have a broad micropore size distribution: in the ultramicropore range of ≤0.7 nm for PC-N1 and PC-N2 samples and in the larger supermicropore range of 1–2 nm for the PC-P sample. All the PC samples also have a multimodal mesopore size distribution in the range of 2–10 nm with the peaks at 3.5 and at 5 nm for PC-N1 and PC-P samples, while PC-N2 sample has pronounced peaks at around 2.5 nm and 5 nm. It can also be seen from Figure 1 that capillary condensation occurs due to the presence of mesopores (2–50 nm) in the materials, which is evidenced by the shape of the hysteresis loop formed by the adsorption and desorption branches of the isotherms. From the PSD curves, it follows that the porous structure of the tested materials is characterized by a polymodal distribution with the peaks in the range of both the micro- and mesopores’ size.

The textural characteristics of the obtained PC samples are shown in Table 1: S_BET_: Brunauer–Emmett–Teller (BET) theory specific surface area; S_QSDFT_, V_QSDFT_ and D_QSDFT_ are the specific surface area, the total pore volume and the average pore diameter, calculated by quenched solid density functional theory (QSDFT) method, respectively; V_BJH_ is the mesopore volume, calculated using the Barrett–Joyner–Halenda (BJH) method. V_DR_ is the volume of micropores, calculated using Dubinin–Radushkevich (DR) method; MBN: methylene blue number; IN: iodine number.

In general, activated carbon obtained from rice husk using KOH as an activating agent has a very large surface area with high meso- and micropore volumes, carbon samples activated with H_3_PO_4_ have also demonstrated relatively high textural characteristics [26].

The calculation of surface-related properties based on S_BET_ alone can be misleading and a better estimation technique is required. It appears that, for typical nanopores, the BET theory often overestimates the total surface area with respect to other determinations, such as a modern sophisticated QSDFT approach, which offers reliable assessment of porous carbon surface area and has been widely used in recent years [31].

According to the calculations of low-temperature nitrogen adsorption data using BET and QSDFT methods, it was determined that the specific surface areas (S_BET_ and S_QSDFT_) and QSDFT total pore volume (V_QSDFT_) of PC samples have the following values: PC-N1: 2690, 2330 m^2^/g and 1.84 cm^3^/g; PC-N2: 1410, 1360 m^2^/g and 0.91 cm^3^/g; PC-P: 1700, 1400 m^2^/g and 1.56 cm^3^/g, respectively.

Due to its smaller molecular size (0.4 nm*^2^* cross-sectional area), iodine can access narrow micropores that are inaccessible to the larger methylene blue molecule, which has a cross-sectional area of 2.08 nm*^2^* and requires larger micropores or mesopores [32]. Indeed, from the data shown in the Table 1 it follows that the values of V_DR_, IN and MBN correlate with one another: V_DR_ for PC-P (0.48 cm^3^/g) < PC-N2 (0.63 cm^3^/g) < PC-N1 (1.01 cm^3^/g), while MBN and IN for PC-P (538 and 960 mg/g) < PC-N2 (621 and 1545 mg/g) < PC-N1 (1571 and 2810 mg/g), respectively.

On the other hand, the values of mesopore volume assessed by the BJH method correlate with average pore diameter calculated by the QSDFT method: V_BJH_ (cm^3^/g) and D_QSDFT_ (nm) for PC-N2 (0.39 and 0.82) < PC-N1 (0.85 and 0.89) < PC-P (1.15 and 1.10), respectively; on the contrary, the ratio of micropore volume to QSDFT total pore volume increases as follows: V_DR_/V_QSDFT_ for PC-P (0.308) < PC-N1 (0.549) < PC-N2 (0.692).

According to thermodynamic calculations [33], it is shown that the net process of carbothermic potassium reduction with the use of KOH is the reaction expressed by the following summary Equation (1):

6KOH + 2C → 2K + 2K_2_CO_3_+ 3H_2_↑ (1)

Nevertheless, the resulting potassium carbonate in a molten state is capable of leaching silica [26] and can be further reduced to potassium, according to the reactions Equations (2) and (3) as follows:

2K_2_CO_3_ + SiO_2_ → K_2_SiO_3_ + CO_2_ (2)

K_2_CO_3_ + 2C → 2K + 3 CO (3)

Factually, overall chemical reactions during the process of carbonized RH (CRH) KOH activation at high temperature can be represented by the following equations:

2KOH + SiO_2_ → K_2_SiO_3_ + H_2_O (4)

H_2_O + C → CO↑ + H_2_↑ (5)

2KOH + 2C → 2K + 2CO↑+ H_2_↑ (6)

**Figure 2:**
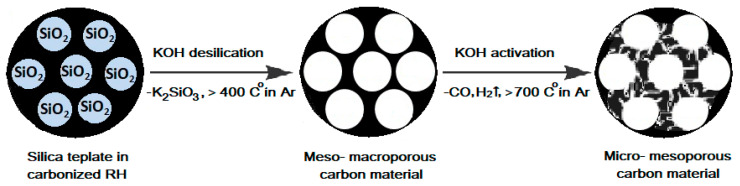
Formation of micropores and mesopores in a carbon matrix during high-temperature alkaline activation. Adapted from [34].

Figure 2 shows a diagram of the mesopore formation due to the leaching of phytoliths (SiO_2_ matrix) from RH during carbonization and micropores as a result of carbothermic reduction and subsequent intercalation of potassium compounds into the interplanar space of a graphite-like structure, which considerably increases distances between the planar fragments of the graphite layer [33].

In a number of studies, the authors found that the majority of micropore formations, the proportion of which decreases with increasing temperature in the range of 700–1000 °C, depend on the duration of the process and the ratio of activated and activating materials [26]. Instead, micropore expansion predominates over the formation of new micropores. This occurs due to the large number of research centers located within the micropores rather than on the outer surface. This pore expansion results in the conversion of the carbon material’s micropores into mesopores. As the degree of combustion decreases, coarsening and, in traditional calculations, merging of mesopores, occur, which ultimately leads to the collapse of the porous structure and a decrease in the specific surface area and total pore volume.

In this regard, the formation of porous space in RH-derived adsorbents occurs due to the following processes:

(1) macropores: during carbonization, due to the preservation of RH’s native structure and subsequent leaching of silica phytolith template from CRH;
(2) mesopores: mainly due to high-temperature leaching of the SiO2 template (Equation (4)), as well as burn-off of the carbon matrix (Equations (5) and (6));
(3) micropores: mainly due to carbothermic reduction reactions of potassium (Equation (3)), as well as leaching of SiO2 (Equation (4)) and burn-off (Equation (5)).

**Figure 3:**
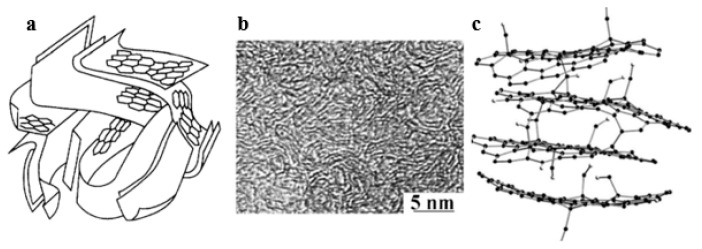
Structure of microporous carbon materials at nanoscale: (**a**): theoretical model of a “crumpled paper ball”, (**b**): high-resolution electron micrograph, (**c**): computer nanoscale model of turbostratic structure layers. Adapted from [31,35].

The meso- and microtexture of PCs is formed by gaps of corresponding sizes between layered carbon structures; micropores in many cases correspond to gaps between turbostratic graphenes. Therefore, micropores in PCs often have a characteristic slit-like morphology. Figure 3 shows a computer model of the turbostratic structure of graphite-like carbon layers [35,36].

The structure of typical of nanoporous carbons from lignocellulosic precursors is represented by pores, typically bounded by sp^2^ hybridization, distorted graphene layers, slit shaped in most cases. Pore shape and distribution depend on the precursor and activation conditions. Distribution of pores is in the 0.5 to 2 nm range. High electron density in graphene layers promotes strong adsorption by van der Waals forces, but it is strongly dependent on pore size and adsorbate molecule size controls heat of adsorption [35,36].

#### 2.1.2. Surface Structure and Morphology and the Elemental Content of the PC Components According to SEM/EDS Analysis

**Figure 4:**
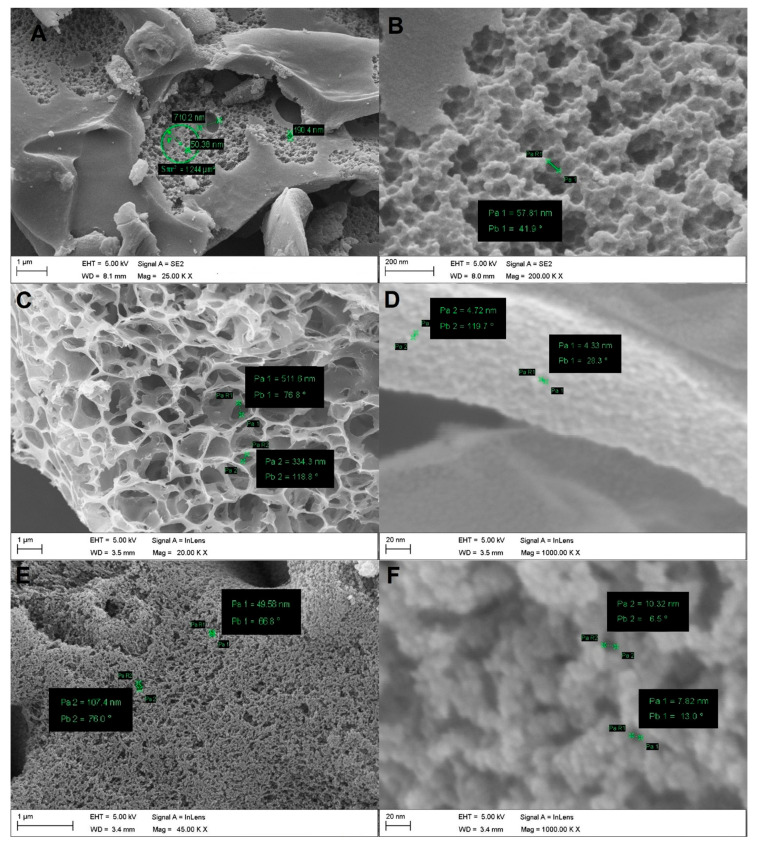
SEM images of the samples: PC-N1 (**A**,**B**); PC-N2 (**C**,**D**) and PC-P (**E**,**F**).

The studies of surface morphology structure (microstructure and porosity) of the obtained PCs were conducted by scanning electron microscopy (SEM) using a Zeiss NTS electron microscope with an accelerating voltage of 5 kV. SEM images of PC samples are shown in Figure 4, where the green lines and circles show the size of the pores.

The SEM image of the PC-N1 sample shown in Figure 4A exhibits channels in porous material—transport macropores. In spite of high-temperature chemical activation with KOH and subsequent oxidative ammonolysis, the PC retained the spongy macrostructure, typical for RH-derived carbon nanomaterials [26]. However, morphological analysis of the SEM image shown in Figure 4B indicates that the surface of the PC-N1 sample underwent a number of changes, e.g., during chemical activation, surface erosion (etching) and the formation of additional mesopores took place due to reactions, shown in Equations (1)–(6); these macropores contributed to the formation of new meso- and micropores in the internal surface. Hence, the interconnected open meso- and microporous structures can promote the mass transfer of phenolic compounds, resulting in the improvement of adsorption capacity [37]. The existence of mesopores and 3D interconnected pore structures might reduce the diffusion resistance.

From the SEM image of the PC-N2 sample, shown in Figure 4C, it can be observed that, despite the high-temperature chemical activation reactions with K_2_CO_3_, represented by Equations (2) and (3), followed by oxidation with HNO_3_ and further high-temperature urea treatment, the material has retained the cellular macrostructure; at higher resolution, it can be clearly seen that the sample contains small mesopores, as shown in Figure 4D.

Typical SEM images of the PC-P sample, obtained by RH activation with H_3_PO_4_ at high temperature and subsequent desilication reaction with KOH or NaOH in aqueous solutions, represented by Equation (4), are shown in Figure 4E,F. Apparently, the sample retained the distinctive morphological structure, typical for RH-derived carbon nanomaterials, despite the harsh chemical treatment, as shown in Figure 4E. At higher resolution, Figure 4F, it is evident that the spongy surface of the PC-P sample is characterized by a well-developed mesoporous structure.

Elemental distribution profiling via energy-dispersive X-ray spectroscopy (EDS) was conducted across the three PC samples. The EDS spectrogram (Figure S1, Supporting Information) indicates that the elemental composition of all samples predominantly comprises light elements, including carbon (C), oxygen (O) and nitrogen (N).

The semi-quantitative content of elements in PC samples was assessed by use of EDS microanalysis and is given in Table 2. In brief, from the data shown in Table 2 it was determined that the carbon and oxygen contents in PC samples have the following values: PC-N1: 92.4%, and 6.7%; PC-N2: 96.1% and 3.0%; PC-P: 92.4% and 3.5%, respectively. Phosphorus content in the PC-P sample is 2.5%; all PC samples have trace amounts of silicon and sulfur of ca. 0.1% each. All PC samples also have a small number of alkaline metals, e.g., potassium contents for PC-N1 and PC-N2 samples are 0.7% and 0.8%, respectively, and the sodium content for PC-P is 1.3%, which indicates incomplete washing due to the presence of acidic groups in PC samples, i.e.,: phenolic and carboxylic groups in PC-N1 and PC-N2 samples and phosphate groups in the PC-P sample, as will be demonstrated later by XPS analysis in Section 2.2.

### 2.2. Surface Chemistry of Modified PCs

#### 2.2.1. FT-IR Spectroscopy of Surface-Functionalized Nanoporous Carbons

**Figure 5:**
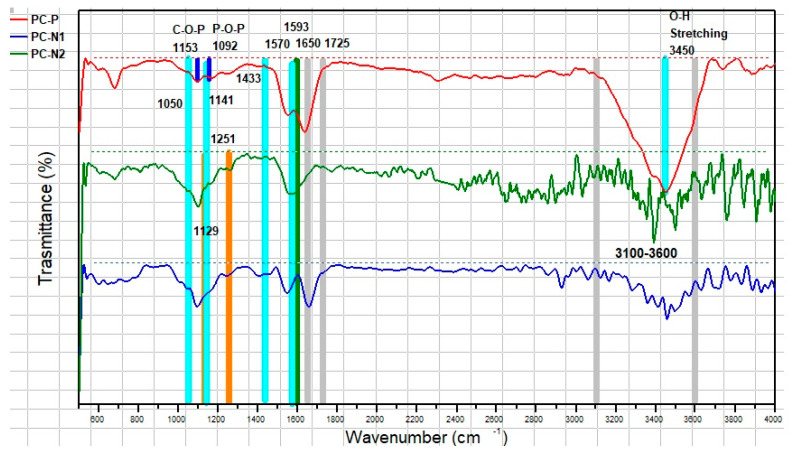
FT-IR spectra of PC-N1, PC-N2 and PC-P samples.

FT-IR spectra of three porous carbons used in this work (PC-N1, PC-N2 and PC-P) are shown in Figure 5.

It has been previously reported that N-doped porous carbons, like PC-N1 and PC-N2, show typical peaks at 3600–3100, 1593 and 1050 cm^−1^ that correspond to the O–H stretching vibrations, a combination of C=C and C=N in-plane stretching vibrations and C–O stretching vibrations, respectively. The broad band in the region of 1725–1650 cm^−1^, observed for PC-N1 and PC-P, can be attributed to (νC=O) stretching vibrations and is absent for PC-N2 due to CO volatilization due to high-temperature treatment during the synthesis, which is in good agreement with XPS data. Skeletal vibrations of aromatic rings (1570 and 1433 cm^−1^) and νC–O of C–OH (1141 cm^−1^), attributed to alcohol and/or carboxyl functional groups, are characteristic of all PC samples and are also in good agreement with XPS data. The pronounced band at 1251 cm^−1^ (νC–N aromatic), present in PC-N1 and PC-N2, is good evidence of the surface N-doping [38].

A broad absorption band in the region 3200–3600 cm^−1^ with the maximum around 3450 cm^−1^ of the PC-P sample’s FT-IR spectrum can be assigned to the O–H stretching mode of phosphoric acid functional groups. The band around 1220–1180 cm^−1^ can be attributed to the stretching of the P=O bond in a phosphate ester, the O–C bond in P–O–C linkage or the P=OOH bond in phosphorus-containing groups. The band around 1700 cm^−1^ is usually caused by the stretching vibration of C=O in ketones, aldehydes, lactones and carboxyl groups; the band around 1600 cm^−1^ is attributed to an aromatic ring or C=C stretching vibration. The band at 1216 cm^−1^ is typically attributed to the C–O bond. The 1153 cm^−1^ band corresponds to the stretching vibration of hydrogen-bonded P=O and C–O stretching vibration in C–P, while the 1092 cm^−1^ band is related to P–O–P stretching [39].

#### 2.2.2. XPS Analysis of Functionalized PC Surface

XPS analysis was performed to gain a deeper understanding of the degree of chemical modification and surface chemistry (decoration) of PCs derived from RH. The results of XPS survey scan analysis giving the atomic percentage of elements are presented in Table 2, in comparison with EDS microanalysis data.

**Figure 6:**
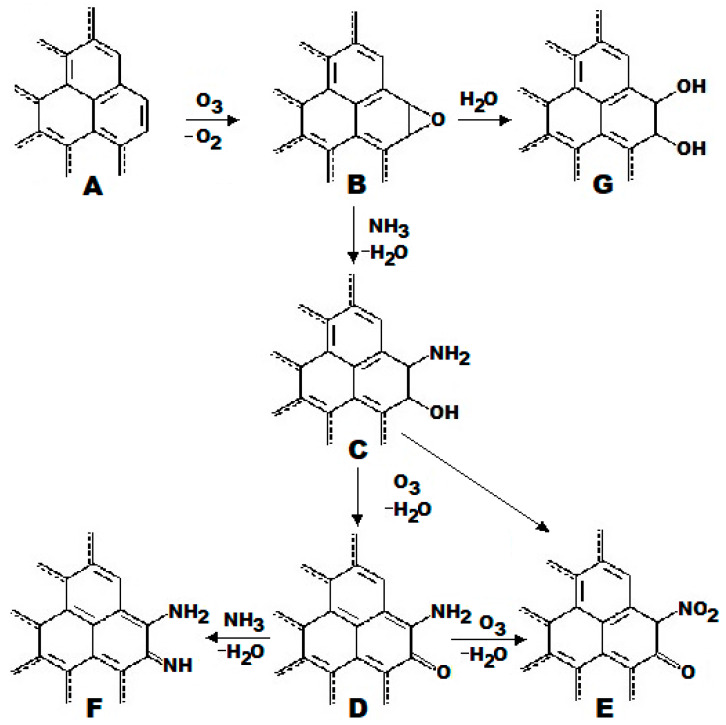
The proposed mechanism of PC surface chemical transformations to form functional groups in the course of ammoxidation reactions to yield PC-N1: (**A**)—fragment structure of graphene edge of PC-N1 precursor (carbonized and KOH activated RH, as shown in Figure 2 and described in Section 3). (**B**)—fragment structure of epoxide (oxirane) functional group, formed due to ozonation of fragment structure A as described in the Section 3. (**C**)—fragment structure of amino phenolic (hydroxylic and amino-) functional groups formed upon nucleophilic attack of fragment structure B with ammonia in the course of ammoxidation (see the Section 3). (**D**)—fragment structure of keto group, derived from oxidized phenolic group (fragment structure C) by an excess of ozone adsorbed on the PC surface. (**E**)—fragment structure of surface nitro group, formed by the ultimate oxidation of amino group in fragment structure D with ozone adsorbed by the PC. (**F**)—fragment structure of aminated carbonyl groups in fragment structure D with excess of gaseous NH3. (**G**)-“catechol-like” fragment structure, created by hydrolysis of epoxide (oxirane) functional group in fragment structure B.

The XPS results show that the amount of carbon in the PC-N2 sample is higher than that of the PC-N1 sample (96.8% vs. 90.9%); the amount of nitrogen in the PC-N2 sample is about the same as in the PC-N1 sample (1.4% vs. 1.3%). On the other hand, the amount of oxygen in the PC-N1 sample is bigger than in the PC-N2 sample (6.7% vs. 1.4%). This discrepancy is expected and can be explained by a difference in synthetic conditions for these two samples.: the PC-N1 sample was oxidized by ozonation in ambient conditions and treated with ammonia at a relatively low temperature of 350 °C, while the PC-N2 sample was obtained by nitric acid oxidation, followed by nitrogenation with urea at a high temperature of 950 °C, and it lost most of the oxygen due to CO volatilization. It also means that the PC-N1 sample is expected to have a considerable amount of oxygen-containing functional groups, as will be demonstrated later by a proposed reaction mechanism of its surface ammoxidation, according to a scheme depicted in Figure 6. The results of XPS and EDX analyses regarding the correlation of the main element content of carbon in PC-N1 and PC-N2 samples and oxygen in PC-N1 are in good agreement.

As follows from the results of elemental analysis of PC samples using XPS and EDS techniques, the contents of particular elements on the surface measured by EDS are somewhat different from those in the bulk measured by XPS. Indeed, in the case of the PC-P sample, the phosphorus content attained by use of EDS is bigger than that by XPS and, vice versa, the oxygen content assessed using EDS is lower than that by XPS. The reason for these discrepancies is not clear; however, it may be related to the difference in the chemical composition of carbons at the surface and in the bulk, since these methods probe materials to different depths.

The presence of sodium in the EDS spectrum of the PC-P sample is indirect proof of functional phosphate groups covalently bound to the carbon surface (Table 2, Figure S1).

Gaseous NH_3_ is the most commonly used reagent for synthesis of N-doped PC, either alone or following preoxidation with an oxidizer, such as concentrated nitric acid via the amination pathway, as well as ammonia–air mixtures treatment via a process called ammoxidation. The nitrogen content introduced depends on the ammoxidation temperature, with the process being most intense during the first hour. Ammoxidation at 350 °C increases basicity of an N-doped PC surface, and the resulting terminal amino groups, not involved in amide formation, remain reactive, exhibiting well-defined protonation to form positively charged aminium groups. In general, the ammoxidation process mildly reduces the pore volumes and surface area of PCs, although subsequent heat treatment can restore PCs’ textural properties. Specifically, N-containing functional amino groups formed in this way are thermally unstable and mainly localized at the edges of the PC surface. To overcome this hurdle, N-containing precursors, such as carbamide or melamine, with defined nitrogen content are used. The temperature of the carbonization process also influences N atoms’ incorporation into the carbon lattice, e.g., at 500–700 °C, nitrogen was found to exist primarily in the state of pyrrolic-N, pyridinic-N, amine and imine, as well as amide groups, while activation above 800 °C transforms nitrogen into quaternary-N and pyridonic-N functional groups [40].

A plausible mechanism of the PC ammoxidation reactions, i.e., involving ozone, reacting with double bonds of unsaturated carbon first, followed by the formed epoxide compound ring opening by ammonia during the amination step, is summarized in the scheme presented in Figure 6.

The mechanism of nitrogen incorporation into the carbon matrix during KOH-activated PC surface ammoxidation via ozonation, hypothesized here, occurs via the Criegee mechanism of O_3_ cycloaddition to the edges of graphite-like fragment structure A (Figure 6A) to form an intermediate metastable fragment structure B followed by amination [41].

According to this mechanism, formation of epoxide groups occurs in the first place to form edge fragment structure B from its precursor (fragment structure A) with subsequent transformation of the unstable three-membered oxirane ring attacked by the strong nucleophilic molecule of ammonia resulting in formation of hydroxylic and amino groups on the carbon surface to produce fragment structure C. Phenolic groups of fragment structure C could be eventually oxidized to keto groups by an excess of ozone adsorbed on the carbon surface to form fragment structure D, leaving amino groups intact in a reducing environment of ammonia. The latter can undergo further transformation in two ways: surface amino groups in fragment structure D can be ultimately oxidized to nitro groups by the remaining ozone adsorbed on the carbon surface to yield structure E; alternatively, the oxygen atoms of carbonyl groups in fragment structure D can be substituted with nitrogen atoms by successive reaction with ammonia to form fragment structure F, the edges of which are decorated with both imine and amino groups. In addition, it is plausible to some extent that the oxirane rings of graphene-like fragment structure B can undergo nucleophilic attack by slowly emerging water molecules, which is accompanied by epoxy ring opening, resulting in the formation of “catechol-like” fragment structure G.

**Figure 7:**
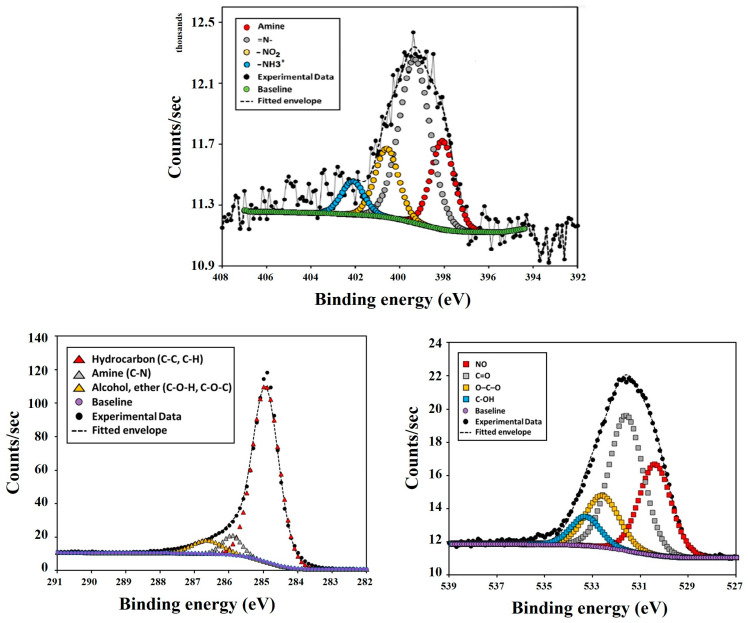
N1s, C1s and O1s XPS high-resolution spectra of PC-N1 sample.

The high-resolution XPS spectra for N1s, C1s and O1s of the PC-N1 sample are presented in Figure 7 (the full XPS spectra is presented in Figure S2)and the relative contents of each functional group calculated from their peak areas are summarized in Table 3.

The peaks of the C1s spectra for the PC-N1 sample with binding energy (BE) at 284.94, 285.93 and 286.64 eV were associated with C–C, C–H (hydrocarbon); C–N (amine) and C–O– (alcohol/ether), respectively. Four evident peaks could be detected in N1s spectra, corresponding to: –C–NH_2_ (amine), 398.09 eV; –C=NH (imine), 399.34 eV; –C–NO_2_ (nitro), 400.59 eV and –C–NH_3_^+^ (aminium), 402.1 eV. The four peaks of O1s spectra with binding energy (BE) at 530.39, 531.6, 532.6 and 533.3 eV were associated with –NO_2_ (nitro) and N-oxide (pyridine); –C=O (carbonyl); O=C–O^−^ (carboxylic); C–OH (phenolic), respectively [41].

**Figure 8:**
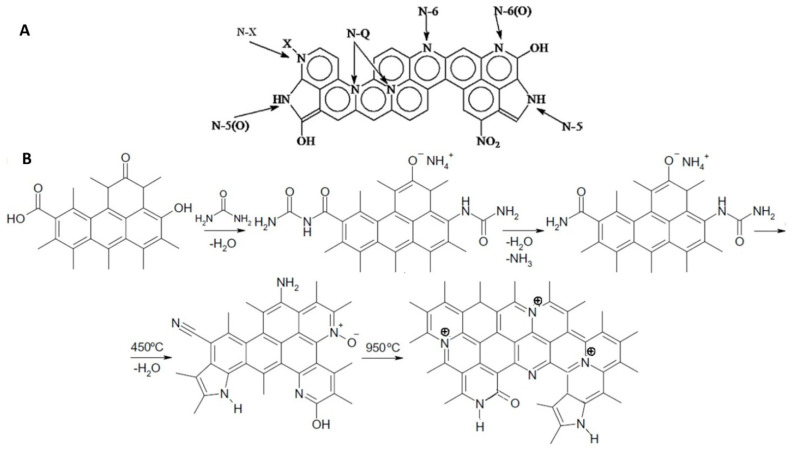
(**A**): Nitrogen forms in porous carbons [42] and (**B**): a scheme of possible chemical transformations of the oxidized PC surface functional groups through reaction with urea at high temperature to yield PC-N2. Adapted from Bandosz et al. [43].

A possible reaction scheme for the incorporation of nitrogen into a carbon matrix during high-temperature treatment of oxidized PC impregnated with urea, leading to the formation of pyridine, pyrrole/pyridone functional groups, is shown in Figure 8 [41,42].

**Figure 9:**
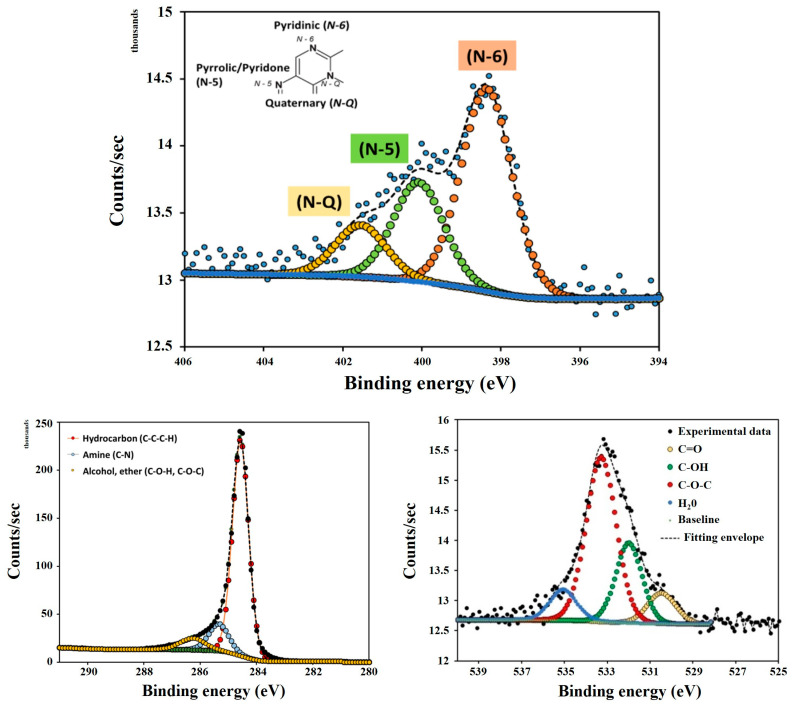
N1s, C1s and O1s XPS spectra of PC-N2 sample.

The high-resolution XPS spectra for N1s, C1s and O1s of PC-N2 sample are presented in Figure 9 and the relative contents of each functional group calculated from their peak areas are summarized in Table 4. N1s and C1 XPS spectra in Figure 9 illustrate the presence of a variety of basic nitrogen groups, i.e., pyridinic, pyrrolic/pyridone and quaternary, incorporated into the carbon matrix of the PC-N2 sample, in accordance with the reaction pathways proposed in Figure 8, while Table 4 represents atomic ratios amongst these main N-species.

The peaks of C1s spectra for in PC-N2 sample with binding energy (BE) at 284.56, 285.34 and 286.26 eV were associated with C–C, C–H (hydrocarbon); C–N (amine) and C–O (alcohol/ether), respectively [44]. Three evident peaks could be detected in N1s spectra, corresponding to: N-6 (pyridinic), 398.36 eV; N-5 (pyrrolic/pyridone), 400.09 eV; N-Q (quaternary), 401.55 eV [43,44]. The four peaks of O1s spectra with binding energy at 530.5, 532.02, 533.31 and 535.1 eV were associated with –C=O (carbonyl) and C–OH (phenol groups); C–O–C (ether groups) and water, respectively [45].

**Figure 10:**
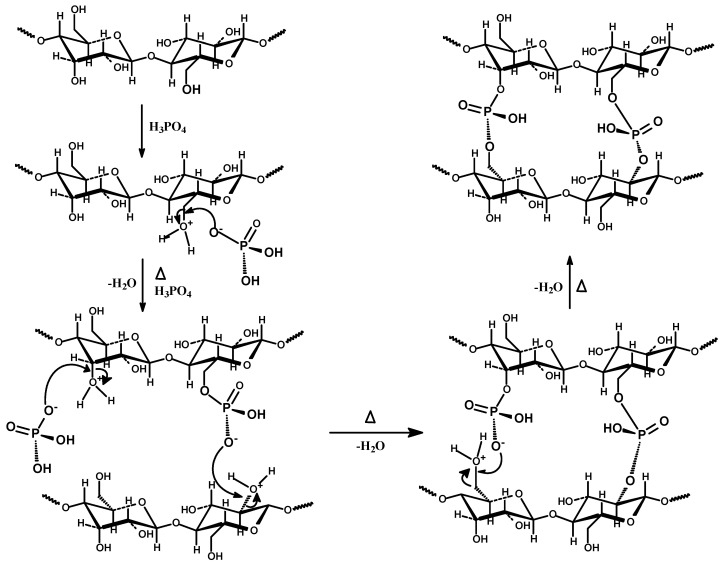
A possible mechanism of cross-linking reactions between cellulose polymer chains with the formation of phosphate bridges via ester bonds in the course of phosphorylation.

Phosphoric acid is a well-known porogen, and when it is used, carbonization reactions occur at significantly lower temperatures compared to standard pyrolysis, which primarily comprises radical processes accompanied by disproportionation reactions, leading to pitch formation since dehydration reactions dominate over radical fragmentation reactions of C–C and C–O bonds, due to acid catalysis. The use of H_3_PO_4_ therefore allows solving two problems: increasing carbon material yield and developing a large specific surface area. In the course of precarbonization, i.e., activation at low temperature within the range of 100–200 °C, vegetable biomass exhibits intense volumetric compression, together with escalated weight loss, primarily due to volatilization of water and carbon oxides, together with low-molecular-mass organic molecules, which are generated through the reactions of dehydration, as well as depolymerization. At elevated temperatures, the mass loss rate decreases rapidly, since phosphoric acid inhibits the lignocellulose decomposition volatile product formation. It was established that, at around 400 °C, the developing carbon matrix starts to expand, which is attributed to cross-linking reactions amongst polymer chains via the ester bonding due to cellulose phosphorylation [25,39], and a probable mechanism of phosphate bridge formation is shown in Figure 10.

With a further temperature rise, condensation and cyclization reactions enhance the aromaticity and polyaromatic fragment size through the cleavage of P–O–C bonds. Following hydrolysis of the (poly)phosphate P–O–P bridges, excess H_3_PO_4_ forms at water-washing and base-leaching steps, and the carbon matrix remains expanded, resulting in a well-developed porous carbon structure [39].

**Figure 11:**
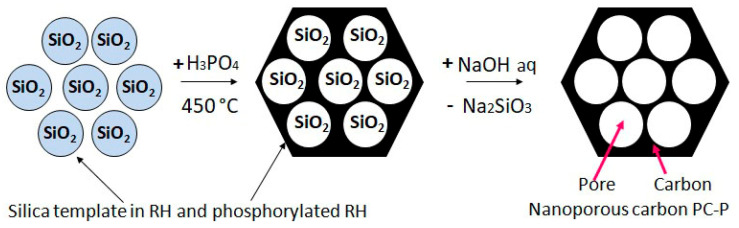
Formation of nanopores in PC-P carbon matrix during RH phosphorylation, followed by leaching silica template in alkaline solution.

The micro- and nanoscale siliceous phytoliths in rice husk act as templates for generating additional meso- and macroporosity. After activation of RH with H_3_PO_4_, the use of alkaline solutions of sodium and potassium hydroxides allows the development of mesoporosity in the PC matrix by leaching SiO_2_ from phosphorylated carbon–silica composite and removal of not only excess phosphates but also the respective water-soluble silicates, as described by reaction Equation (4) and the scheme illustrated in Figure 11.

**Figure 12:**
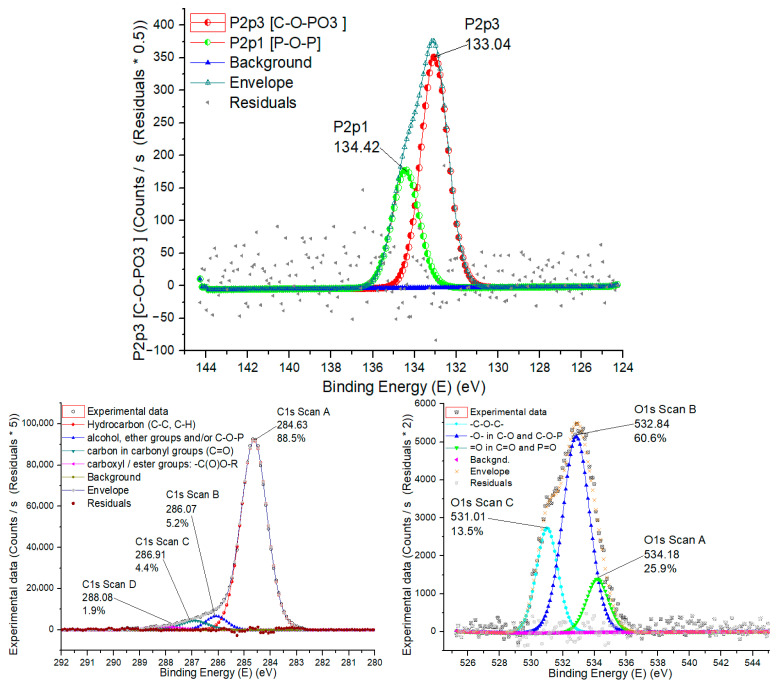
P2p, C1s and O1s XPS spectra of PC-P sample.

The high-resolution XPS spectra for P2p, C1s and O1s of the PC-P sample are presented in Figure 12 and the relative contents of each functional group calculated from their peak areas are summarized in Table 5. P2p and O1 XPS spectra in Figure 12 illustrate the general picture of common phosphorus-containing groups, i.e., C–O–PO_3_ groups in (pyro)phosphates (PO_4_ tetrahedra), phosphorus bound to oxygen in (P–O–P), etc., while Table 5 represents atomic ratios amongst these main P-moieties.

P2p XPS spectra of the PC-P sample are given in Figure 12, reflecting the general picture of the presence of the main phosphorus-containing functional groups, namely: anchor phosphate (P–O–C) groups embedded in the carbon matrix of the PC-P sample adsorbent, as well as polyphosphate (P–O–P) groups.

The peaks of C1s spectra for the PC-P sample with binding energy (BE) at 284.63, 286.07, 286.91 and 288.08 eV were associated with C–C, C–H (hydrocarbon) [46]; C–O–P (and/or alcohol, ether groups); C–O (carbon in carbonyl groups) and (carboxyl and/or ester groups), respectively. Two evident peaks could be detected in P2p spectra, corresponding to: C–O–PO_3_ ((pyro)phosphates (PO_4_ tetrahedra)), 133.04 eV; P–O–P (metaphosphates), 134.42 eV. The three peaks of O1s spectra with binding energy (BE) at 531.01, 532.84 and 534.18 eV were associated with =O (in carbonyl), C=O and P=O (carboxyl and phosphates); C–O and C–O–P (singly bonded oxygen –O–) and C–O–C (ether groups), respectively [47].

### 2.3. Evaluation of the Surface-Modified Nanoporous Carbon Adsorbent Cytotoxicity

**Figure 13:**
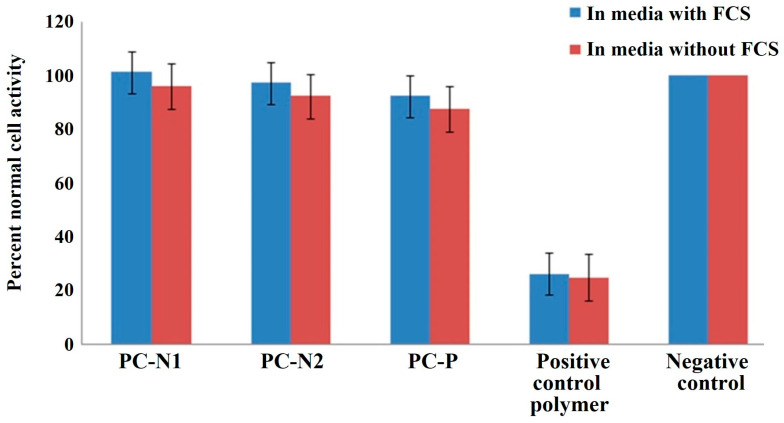
Percent normal cell viability measured using the MTS assay on 50% PC extracts in media with or without FCS, compared to a positive control (mean n = 3 (6 samples), ±sem).

**Figure 14:**
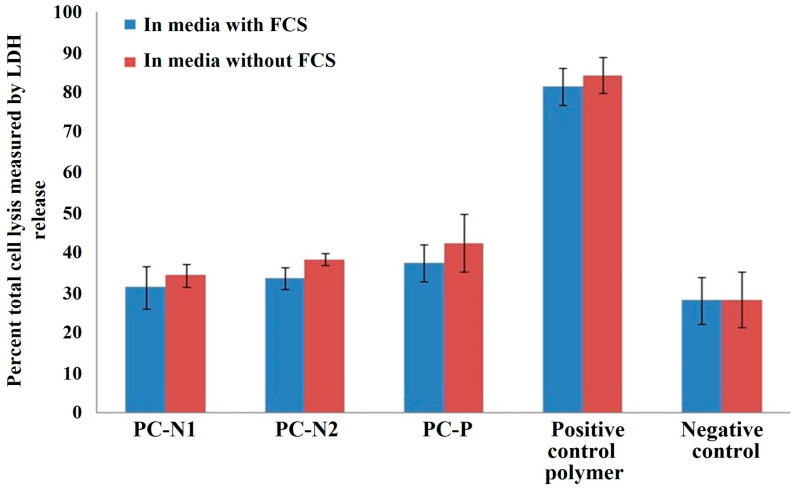
Percent cell lysis measured using the LDH assay on 50% PC extracts in media with or without FCS, compared to a positive control (mean n = 3 (6 samples), ±sem).

The MTS results (Figure 13) indicate all three types of PC show normal cell viability percentages comparable to the negative control media only. Similarly, the LDH results (Figure 14) show cell lysis percentages comparable to the negative control media only. There is no reduction in % normal cell viability or % cell lysis between the 50% PC extraction into media with or without FCS when compared to the negative control. However, there is a significant difference (Student *t*-test, *p* < 0.01) from the 50% positive control polymer extract which showed only ~25% normal cell activity (MTS) and ~85% cell lysis (LDH). The 50% extracts were used to assess PC cytotoxicity as the 100% extracts showed reduced viability and increased cell lysis compared to the negative control. This finding followed our previous study with activated porous carbon [48] which initially demonstrated an inhibition of colony formation and an apparent cytotoxic effect but was determined to be a result of protein and ion adsorption by the large surface area and adsorbent nature of activated carbon in comparison to the small surface area of the positive control polymer. This starved the cells of nutrients and gave erroneous results. In another study it was revealed that alterations in culture media such as protein adsorption by test materials can influence the outcome of cytotoxicity evaluation and that there is a need for precise standardization of testing procedures to encompass diverse materials such as adsorptive materials [49]. Our findings for the 50% PC extracts demonstrated that the PC did not cause any changes to the cell viability or cell lysis as measured by the MTS and LDH assay and appeared to be non-toxic to a V79 cell line under the conditions used and thus may be considered non-cytotoxic to cells and warrant further investigation for hemoadsorption applications.

The cytotoxicity measurement of the PC-N1 PCN-2 and PC-P samples using the modified MTS and LDH methods utilizing 50% extracts demonstrated that the PCs did not cause any changes to the cell viability or cell lysis and thus could be considered non-cytotoxic to cells.

### 2.4. Batch Adsorption Studies of Phenolic and Indolic Compounds

#### 2.4.1. Kinetic Studies of Modified Nanoporous Carbon Adsorbents

**Figure 15:**
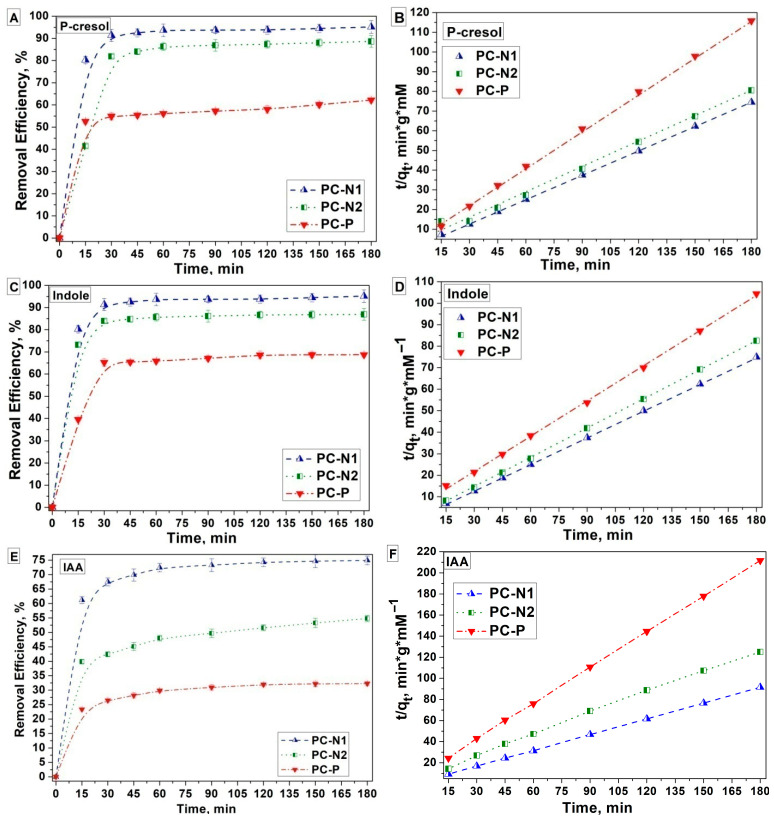
The effect of contact time on the removal rate of p-cresol (**A**), indole (**C**) and IAA (**E**) and the corresponding linearized pseudo-second-order kinetic models for p-cresol (**B**), indole (**D**) and IAA (**F**) adsorbed by PC-N1, PC-N2 and PC-P samples. Experimental conditions: adsorbent dosage 2 mg/mL; the initial concentrations of 5 mM for p-cresol, indole and IAA; pH 7.4; temperature 300 K.

The effect of contact time on the removal rate of p-cresol, indole and IAA and the corresponding linearized pseudo-second-order kinetic models for p-cresol, indole and IAA adsorbed by PC-N1, PC-N2 and PC-P samples are shown in Figure 15. The linearized pseudo-first order kinetic models graphs are shown in Figure S3. In the gastrointestinal tract (GIT), the values of pH range from highly acidic in the stomach (within range of 1.5–3.5) to around 6 in the duodenum and small intestine, rising to 7.4 in the ileum, falling to 5.7 in the cecum and finally achieving the value of 6.7 in the rectum. Accordingly, phosphate-buffered saline (PBS) with pH 7.4 to simulate intestinal fluid (SIF) was used in this work.

The highest uptake capacities were obtained for ammoxidized PC-N1 (1.97 mmol/g for IAA, 2.43 mmol/g for p-cresol and 2.42 mmol/g for indole) or “urea-nitrified” PC-N2 (1.44 mmol/g for IAA, 2.24 mmol/g for p-cresol and 2.19 mmol/g for indole), whilst for phosphorylated PC-P the values were 0.85 mmol/g for IAA, 1.60 mmol/g for p-cresol and 1.74 mmol/g for indole.

In order to understand the kinetic behavior of phenolic and indolic PBUT precursors adsorbed by the PC samples, two kinetic models—the pseudo-first-order and pseudo-second-order models—were investigated, using the corresponding equations in the linear form. The pseudo-second-order model fitted the experimental data better, with high coefficients of determination (R^2^ > 0.999), as compared with the pseudo-first-order model. The kinetic data of all three carbons were best fitted (higher R^2^) by the pseudo-second-order model (Table 6).

In vitro adsorption studies of the PCs involving the investigated phenolic and indolic compounds demonstrated that some RH-derived PCs, modified with functional groups, can effectively reduce clinically relevant levels of PBUT precursors.

A comparison of various biomass-derived PC adsorbents for removal of phenolic and indolic substances, in terms of adsorption capacity together with their textural characteristics, is presented in Table 7. While direct comparison may be limited by differences in experimental conditions and synthetic approaches, it provides an overall perspective regarding the performance of the prepared PC sorbents.

Carbon-based materials are widely used adsorbents for the removal of p-cresol from water media. Zhu et al. used NaOH-activated carbon prepared from coconut shells for the removal of high concentrations of p-cresol from water. In their study they found that an adsorbent with S_BET_ of 520 m^2^/g and V_tot_ of 0.31 cm^3^/g was able to adsorb up to 2.37 mmol/g of p-cresol. In the work of Sabri et al. the corn silk carbon was prepared and activated by physical and chemical activation using CO_2_ and KOH further applied for p-cresol removal. During the experiments it was found that chemically activated samples predominate under the CO_2_-activated adsorbent due the more developed surface area and pore volume and 4.4 and 3.7 mmol/g of p-cresol removal were reached, respectively.

Mitome et al. synthesized ordered porous carbons from resorcinol–formaldehyde and Pluronic F127 copolymer at different ratios with and without KOH activation for the removal of indole from buffer solution [60]. It was found that the KOH-activated sample with a larger surface area and higher porosity values showed the highest removal capacities of indole (2.56 mmol/g), while the non-activated sample showed 10 times less removal. The KOH-RF-F127 (3) sample with mesopore volume of 0.29 showed removal results close to those of the commercial AST-120 carbon which also has V_meso_ of 0.3 cm^3^/g. From those results it can be concluded that mesopores play a key role in the removal of indole from aqueous media. In another study, Xue et al. synthesized N-modified mesoporous carbon materials by carbonization at various temperatures of polymer based on 2,4,6-tris(chloromethyl)mesitylene and ethylenediamine [57]. The results of indole removal by these adsorbents reveal that a higher carbonization temperature of 800 °C gave the largest surface area and highest porosity values (2166 m^2^/g and 0.94 cm^3^/g) but did not show the highest adsorption values (6.36 mmol/g). The adsorbent NDPC-700 showed the most developed mesoporous structure which directly influenced the removal rate and showed a maximum removal capacity of 7.35 mmol/g.

For indole-3-acetic acid removal from aqueous media, Liu et al. utilized N-containing porous carbon with a 328 m^2^/g surface area and 0.12 cm^3^/g micropore volume [61]. Using this adsorbent, it was possible to remove up to 1.19 mmol/g of IAA. The influence of micropores on the removal was also confirmed by Shannon et al. They tested Carbotech activated carbon and Starbon materials carbonized at various temperatures [62]. It was reported that higher temperatures stimulated the micropore development and it was shown that Starbon A800 with V_micro_ of 0.17 cm^3^/g showed 1.34 mmol/g removal of IAA, while Carbotech AC (V_micro_: 0.24 cm^3^/g) reached maximum IAA adsorption capacity of 1.79 mmol/g. Some data on the removal of the uremic toxins studied are presented in Table 7.

#### 2.4.2. Comparative Analysis of Modified PC Adsorption Capacity in Connection with Surface Structural Characteristics

Combined with the FT-IR and XPS results, these findings suggest that both physical and chemical interactions may occur between the phenolic or indolic adsorbates and the prepared PCs. Comparative analysis of modified PC adsorption capacity in connection with their surface structural characteristics and some properties of the phenolic and indolic molecules is shown in Table 8.

When it comes to assessment of adsorption values, a comparative analysis of physicochemical characteristics exhibits correlation amongst them, but not always, e.g., descriptors aryl (phenyl or indolyl) hydroxides vs. respective aryl acetates; the oxygen state in aryl (phenyl or indolyl) hydroxide anions is very different than in respective carboxylate anions; pKa and solubility values of Ar–CH2–OH vs. Ar–O– vs. Ar–COO–, etc.

As follows from Table 8, the solubility of indole-3-acetic acid (IAA) is 8.56 mmol/L, which is much lower than that of acetic acid (miscible with water), phenol (880 mmol/L), benzyl alcohol (396.7 mmol/L) and p-cresol (198.8 mmol/L) and even lower than that of indole (30.29 mmol/L), benzoic acid (28.17 mmol/L), benzene (22.92 mmol/L) and phenylacetic acid (12.71 mmol/L). Moreover, the IAA pKa value of 4.54 is much lower than that of indole (pKb = 17.6), benzyl alcohol (15.4), p-cresol (10.3) and phenol (9.99) and is comparable to those of acetic (4.76), phenyl acetic (4.31) and benzoic (4.2) acids. At the same time, the values of IAA molar mass (175.18 g/mol) are about one to three times larger than those of phenylacetic acid (136.15 g/mol), indole (117.15 g/mol), p-cresol (108.14 g/mol), benzyl alcohol (108.14 g/mol), phenol (94.11 g/mol), benzene (78.11 g/mol) and acetic acid (60.05 g/mol); the cross-sectional area of IAA (~0.48 nm2) is about 1.5 times larger than those of indole (~0.3 nm^2^) and p-cresol (~0.29 nm^2^). As was mentioned before in Table 1 (Section 2.1.1), the ratio of micropore volume to QSDFT total pore volume decreases in the following order: V_DR_/V_QSDFT_ for PC-N2 (0.692) > PC-N1 (0.549) > PC-P (0.308) samples.

Since the iodine number (IN) reflects the amount of smaller micropores (ultrapores), while the methylene blue number (MBN) is proportional to the amount of larger micropores (supermicropores) and smaller mesopores, the IN/MBN ratios for the PCs reflect their relative performance. Indeed, the q_t_/V_DFT_ ratios correlate with IN/MBN ratios as well, since most of these compounds’ molecules are adsorbed in micropores. However, the q_t_/V_DR_ ratio does not follow this trend for indole adsorption on the PC-P sample, probably because slightly basic indole has a nitrogen group and does not have oxygen functional groups, which can interact with slightly acidic surface functional phosphate groups of PC-P via the Lewis acid–base interaction mechanism.

According to the results of adsorption studies (Table 8) in relation to the PBUT precursors (p-cresol, indole, IAA), it was established that sample PC-N1 has the highest sorption capacity. Its textural characteristics correlate with the adsorption capacity for p-cresol (2.43 mmol/g) and indole (2.42 mmol/g). The sample exhibited a lower adsorption capacity for IAA (1.97 mmol/g) but it was comparatively higher than for the samples PC-N2 (1.44 mmol/g) and PC-P (0.85 mmol/g). The PC-P sample has the lowest adsorption capacity in relation to all the PBUT precursors studied, despite the high values of such textural characteristics, with S_BET_ of 1700 m^2^/g, S_QSDFT_ of 1400 m^2^/g and V_QSDFT_ of 1.56 cm^3^/g compared to the PC-N2 sample’s S_BET_ of 1410 m^2^/g, S_QSDFT_ of 1360 m^2^/g and V_QSDFT_ of 0.91 cm^3^/g.

According to the data from Table 8 regarding acidity vs. basicity, the PC-N1 sample demonstrated a slightly acidic pH_pzc_ value of 5.71, which may be explained by the difference in the total amount of acidic (carboxylic –COO– and phenolic Ph–C–OH; 2.9 At.%. in total) and basic (aminium–C–NH_3_^+^, amine –C–NH_2_, imine –C=NH; 1.1 At.%. in total) groups, shown by XPS results in Table 3; nonetheless, according to Boehm titration results, the number of acidic groups is 1.36 meq/g, while the number of basic groups is 1.90 meq/g. The PC-N2 sample presented an alkaline pH_pzc_ value of 8.78, which may be explained by the difference in the total amount of slightly acidic phenolic (Ph–C–OH, 0.8 At.%. in total) and basic (quaternary, pyridinic and pyrrolic/pyridone, 1.4 At.%. in total) groups, shown by XPS results in Table 4; indeed, according to Boehm titration results, the amount of acidic groups is 0.9 meq/g, while the amount of basic groups is 3.4 meq/g. Regarding the PC-P sample, it exhibited a slightly alkaline pH_pzc_ value of 6.14, due to the presence of sodium, according to the EDS spectrum (Table 2, Figure S1, Supporting Information), which is indirect proof of functional phosphate groups covalently bound to the carbon surface. The total amount of –C–O–PO_3_^−^ groups, shown by XPS results (Table 5), is 3.4 At.%; indeed, according to Boehm titration results, the number of acidic groups is 3.83 meq/g.

**Figure 16:**
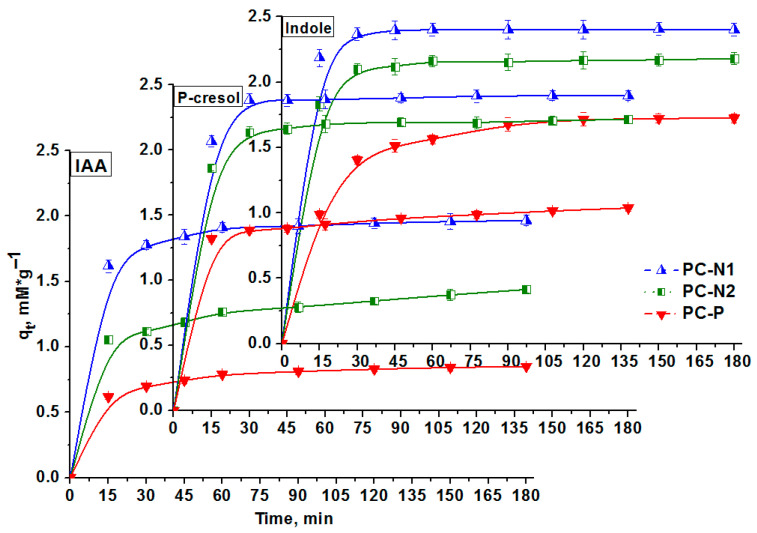
P-cresol, indole and IAA batch adsorption profiles for porous carbons PC-N1, PC-N2 and PC-P over time course (3 h).

P-cresol, indole and IAA batch adsorption profiles for porous carbons PC-N1, PC-N2 and PC-P over 3 h are shown in Figure 16. Indeed, as can be clearly seen from Table 8 and Figure 16, a monotonic decrease in the normalized adsorption amount of indole, and especially IAA, takes placed with an increase in the pore width, which indicates that narrower micropores work as more effective adsorption spaces for the indole molecules.

**Figure 17:**
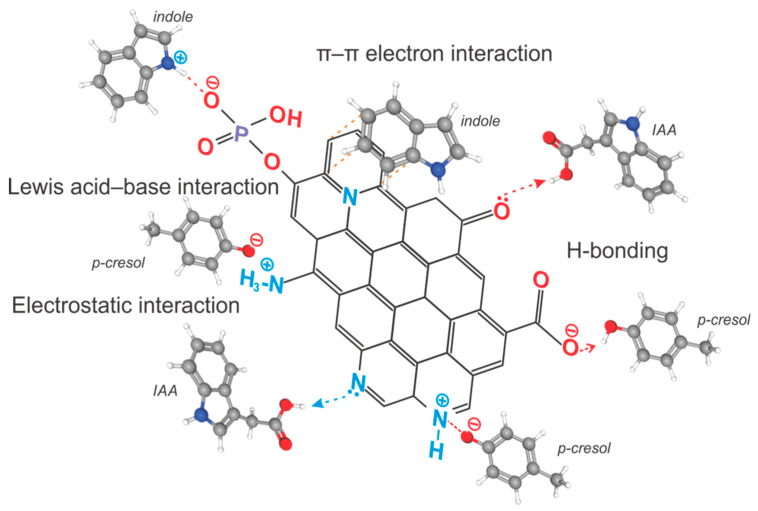
Phenolic and indolic molecule adsorption mechanism by N- and P-doped PCs.

For PC-N1, 80.24% and 95.14% of p-cresol and 87.28% and 96.05% of indole were adsorbed within 15 and 180 min, respectively. For PC-N2, 41.45% and 88.64% of p-cresol and 73.19% and 86.9% of indole were adsorbed within 15 and 180 min, respectively. Steric hindrance and high acidity of both IAA adsorbate and PC-P adsorbent oxygen groups seem to be responsible for the respective adsorption profiles, as demonstrated in Figure 16 and will be discussed below in terms of mechanism (Figure 17). It was reported that there is diffusional hindrance of polar compounds in PCs’ narrow pores and abundant surface oxygen functional groups. Both pore size distribution and surface chemistry of a PC strongly affect indole’s adsorption capacity and adsorption rate. Effective indole removal from aqueous media requires large-surface-area PCs having narrow micropore size with an optimum of approximately 0.70 nm [63]. Additionally, the presence of acidic oxygen functional groups affects the adsorption rate through adsorbate–surface interactions. The primary mechanism of phenol adsorption is filling of micropores smaller than 1.4 nm, with enhanced adsorption attributed to dispersive and repulsive interactions induced by oxygen-containing functional groups [63,64].

The incorporation of N atoms into carbon lattices with large surface areas increases electron donor/acceptor properties, since nitrogen has an additional free electron pair, which can be protonated in acid and hence attract negatively charged ionic species, such as phenolate and acetate anions.

For PC-P with larger amounts of oxygen-containing phosphate functional groups, the p-cresol, indole and especially IAA adsorption capacity values were found to be lower (Figure 16) and the adsorption rates were slower (Figure 15) than those of PC-N2 with less oxygen content, suggesting that PC-P oxygen-containing functional groups strongly interacted with oxygen-containing phenolate and carboxylate anionic groups of p-cresol (–O^−^) and especially IAA (–COO^−^) and, to a lesser extent, with polar indole molecules and thus hindered and delayed further adsorptions of indole to deeper pore spaces.

N-doped PCs with a high degree of aromatization exhibit higher adsorption capacities for phenols. This phenomenon is primarily attributed to π–π interactions between the phenol aromatic ring and the PC surface graphene layers, which is a driving force for selective phenol removal from aqueous solutions [40].

The elimination of organic compounds by functionalized PCs cannot be justified by a single, simplistic mechanism. Organic xenobiotics, in contrast to ions of toxic metals, are typically more nucleophilic due to their electron-rich molecular structures, allowing N-doped and P-doped PC samples to facilitate sorption primarily through both π–π aromatic stacking and Lewis acid–base interactions. Additionally, hydrogen bonding (H-bonding) and electrostatic interactions contribute to the adsorption process. H-bonding is a certain type of dipole–dipole attraction and is a special case of donor–acceptor bond formation. Proton donors are electronegative atoms, such as O, N or F, that covalently bond to hydrogen, like in a hydroxyl group, while proton acceptors are other electronegative atoms with single electron pairs, like the oxygen atom in a carbonyl group. Functional amino, hydroxyl or carboxyl groups on the surface of N-doped PCs can act as hydrogen donors or acceptors, enabling immobilization of organic contaminants via H-bonding by way of chemosorption. On the other hand, electrostatic interactions arise from the attraction between oppositely charged ionizable functional groups on a PC surface and those on organic xenobiotics. Like in the case of toxic metal ion adsorption, the efficiency in removing organic compounds by a PC via electrostatic interactions is also pH-dependent. Finally, N-doping of a PC’s graphene surface aromatic rings generates positively charged sites (holes), which can attract electron-rich organic molecules and that is because of electronegativity differences [65]. Despite the fact that the exact mechanisms of the nitrogen and phosphorus doping effect on modified PCs remain not fully understood, N-doped PC samples have demonstrated significant potential in adsorption applications. Based on these findings, the possible adsorption mechanisms of phenolic and indolic molecules on PCs are illustrated in Figure 17.

The adsorption of acidic organic species onto basic, N-containing functional groups primarily occurs through an ion-exchange mechanism involving proton displacement. PCs’ nitrogenation enhances their basicity, which is advantageous for both wastewater purification and enterosorption applications. In general, the PCs’ basicity is attributed to electron delocalization and electron-donating functional groups which act as Lewis bases.

The incorporation of nitrogen into a PC markedly enhances its surface polarity, thereby improving its adsorption affinity to polar adsorbates. Furthermore, as shown in Figure 17, the adsorption of phenolic and indolic compounds can also be influenced by electrostatic interactions of acidic p-cresol and particularly IAA species with the PC surface basic functional groups, such as C=O, pyridinic N, as well as by π–π dispersion interactions between the aromatic benzene and indole rings and N-doped graphene basal planes [44].

## 3. Materials and Methods

### 3.1. Materials

RH was collected from the rice fields of the Bakanas area, Almaty Region, Kazakhstan. Potassium hydroxide (KOH, 95%), nitric acid (HNO_3_, 65–68%), potassium carbonate (K_2_CO_3_ 95%), concentrated PA (H_3_PO_4_, 85%), urea (carbamide 96%) and sodium hydroxide (NaOH, 96%) were purchased from Jiangxi Ganyi Technology Co., Ltd. (Yichun, China). IAA (99%), p-cresol (99%) and indole (99%) (Sigma-Aldrich, Darmstadt, Germany) were used in all adsorption experiments without additional purification.

### 3.2. Synthesis of Surface-Modified Nanoporous Carbon Adsorbents

**Figure 18:**
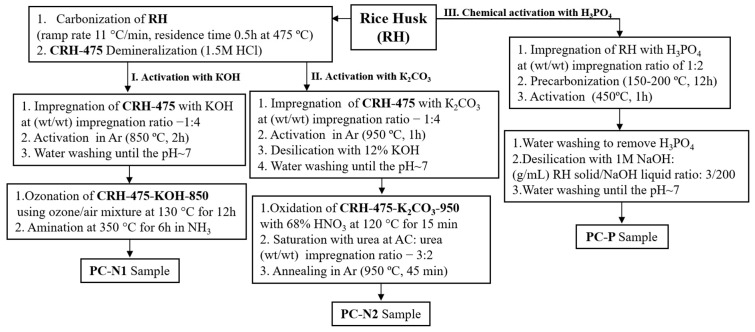
Schematic presentation of synthesis routes and conditions of obtained adsorbents.

Adsorbents used in this study were obtained from rice husk via a procedure described elsewhere [23,26,66]. The adsorbents were synthesized by in situ activation of RH or carbonization followed by activation and modification. The detailed experimental conditions of synthesizing all adsorbents are summarized in Figure 18.

The specific details of the techniques of PC-N1 sample synthesis (via route I. Activation with KOH) are described in [25], while the key methodology and conditions of PC-N2 sample preparation (via route II. Activation with K_2_CO_3_) are given in [66]. The evaluated core parameters for production of the PC-P sample (via route III. Chemical activation with H_3_PO_4_) are specified in [67].

#### 3.2.1. RH Carbonization

For postactivation and modification, 150 g of preliminary washed and dried RH was pyrolyzed at 475 °C in a spherical rotary steel reactor equipped with a capillary tube for water supply with temperature ramp rate of 11 °C/min for 30 min. The water feed rate was set at 100 mL/h with a peristaltic pump. The carbonized rice husk with total yield of ~41% thus obtained was labeled CRH-475 and used for further activation steps.

#### 3.2.2. Activation of Carbonized Rice Husk with KOH

The CRH-475 sample was mixed with potassium hydroxide pellets (KOH ≥ 85.0 wt.%) in a 1:4 wt./wt. ratio, placed into a cylindrical steel reactor of a vertical electric furnace and activated at 850 °C for 2 h in an Ar atmosphere. After the activation step the adsorbent was thoroughly washed with hot distilled water until pH 7–8 and dried in an oven at 110 °C to constant weight. The final yield of the resulting sample was~18%. The sample was labeled as CRH-475-KOH-850.

#### 3.2.3. Oxidative Ammonolysis of Activated Carbon

A mild-temperature ammoxidation of the CRH-475-KOH-850 sample was carried out in a quartz tube reactor inside a horizontal electric furnace. The sample was placed in a quartz boat and oxidized with an ozone/air mixture at 130 °C for 12 h (at gas flow rate of 1 L/min), immediately followed by the reaction with gaseous ammonia in the same reactor (one-pot synthesis) at 350 °C for 5 h (at gas flow rate of 5–6 mL/min) to yield 93.1 wt.% of the ammoxidized product labeled as PC-N1.

#### 3.2.4. Activation of Carbonized Rice Husk with K_2_CO_3_

For the chemical K_2_CO_3_ activation, CRH-475 was mixed with K_2_CO_3_ in a 1:4 wt./wt. ratio and placed in a cylindrical steel reactor inside a vertical electric furnace at an activation temperature of 950 °C for 1 h in an Ar atmosphere. The resulting CRH-475-K_2_CO_3_-950 adsorbent was washed with distilled water until pH ~7, to achieve a complete removal of potassium silicate from the carbonized sample, and then air-dried overnight at 110 °C.

#### 3.2.5. Oxidation of CRH-475-K_2_CO_3_-950 Using Concentrated HNO_3_

CRH-475-K_2_CO_3_-950 was oxidized by mixing concentrated (63%) nitric acid in a 1:2 mass:volume ratio and heating at 120 °C for 15 min in a glass beaker. During the oxidation the reddish-brown nitrogen dioxide was volatilized violently and terminated by addition of cold distilled water. Upon washing off the acid using hot distilled water until pH 6 and drying to constant weight, the oxidized carbon product CRH-K_2_CO_3_@950-HNO_3_ with a total yield of 96% was obtained.

#### 3.2.6. N-Functionalization of CRH-475-K_2_CO_3_-950 with Urea

The CRH-K_2_CO_3_@950-HNO_3_ was mixed with urea in its alcohol solution form at the (wt./wt.) impregnation ratio of 3:2, dried, placed into a quartz boat and finally annealed at 950 °C in a quartz tube reactor of a horizontal electric furnace under Ar flow (at a rate of 5 L/h) for 45 min. The hot quartz tube reactor was quickly removed from the furnace, rapidly cooled to room temperature and the final carbon product labeled as PC-N2 was collected from the quartz boat and dried in an oven at 105 °C to constant weight.

#### 3.2.7. Activation of Rice Husk with H_3_PO_4_

A cylindrical quartz reactor with a mixture of bare rice husk with 85% phosphoric acid at the (wt./wt.) RH:H_3_PO_4_ impregnation ratio of 1:2 was baked in an oven at 200 °C overnight. A precarbonized sample was activated in a vertical cylindrical furnace at 450 °C for 1 h at a heating ramp rate of 5 °C/min. Activation was conducted in a self-generated atmosphere. The activated material was washed a few times with hot water to remove residual acid. For the etching of the silicates from the structure of the activated carbon, the synthesized sample was boiled for 30 min in 1M NaOH. Then, the sample was washed with distilled water until pH 7 was reached. The obtained adsorbent was labeled as PC-P.

### 3.3. Physicochemical Characterization of PC Adsorbents

The morphology of obtained adsorbents was studied with QUANTA 3D 200i (FEI, Long Island, NY, USA) scanning electron microscopy (SEM) equipped with an energy dispersive X-ray spectrometer (EDS) at an accelerating voltage of 20 kV. All samples were preliminarily coated with a 4-nm-thick layer of platinum using a Q150TES (Quorum Technologies, East Sussex, UK) coater. X-ray photoelectron spectroscopy (XPS) was performed using an ESCALAB 250 Xi system (Thermo Scientific, Waltham, MA, USA) with a monochromated X-ray source of Al Kα X-rays, hemispherical electron energy analyzer, magnetic lens and video camera for viewing the analysis position. Wide and narrow spectra of N 1s, C 1s, O1s, P 2p and Si 2p were acquired from three separate areas for each sample. The XPS data was corrected and analyzed using Thermo Avantage software (Version 5.952) with a smart background. For determination of functional groups, FT-IR spectra of PC samples were recorded in the range of 4000–400 cm^−1^ with the resolution of 4 cm^−1^ using a Cary 600 Series FT-IR spectrophotometer (Agilent Technologies, Santa Clara, CA, USA) equipped with an ATR module. For low-temperature nitrogen adsorption analysis (LTNA, Union, WA, USA), the PC samples were degassed for 3 h at 200 °C before the analysis was carried out. The carbon adsorbents were analyzed and nitrogen adsorption/desorption isotherms determined with an Autosorb-1 analyzer (Quantachrome, Hook, UK) in the range of relative pressures from 0.005 to 0.991. The data was analyzed using “AqiQwin 3.01” data analysis software. The specific surface area was calculated using the BET method; the pore size distributions, surface area and pore volumes of the materials were calculated using the DFT slit/cylindrical pore model recommended for activated carbon [10].

### 3.4. Analysis of the Adsorbent Cytotoxicity

The quantitative determination of cytotoxicity of PC extracts was evaluated using the MTS and LDH assay for cell viability/lysis adapted in house for suitability to assess adsorbent materials. The 3-(4,5-dimethylthiazol-2-yl)-5-(3-carboxymethoxyphenyl)-2-(4-sulfenyl)-2H-tetrazolium (MTS) assay is a colorimetric method for assessing cell viability, as the cellular oxidoreductase enzyme can reduce colored tetrazolium dye (formazan) and reflect the number of viable cells present. The lactase dehydrogenase (LDH) assay detects the stable LDH enzyme common in all cells and is measured when cell membranes are no longer intact (cytolysis). Materials (0.1 g) were extracted into media (1 mL) with or without fetal calf serum (FCS) at 37 °C (5% CO_2_) for 24 h, with 50–100% extract solutions measured to circumvent the large surface area and ability of the PC sorbents to adsorb essential nutrients from the media and influence cell growth. Extracts were filtered using a 0.45 µm preconditioned surfactant-free cellulose acetate syringe filter (Whatman, Maidstone, UK) to sterilize the samples and 100 µL (in triplicate) incubated at 37 °C (5% CO_2_) for 24 h with V79 cells (Chinese hamster lung, male, ECACC No. 86041102) preseeded into 96-well plates. The experiment was performed on three separate occasions in duplicate. Results were compared to a negative control (no material extract) and a positive control of a PVC polymer containing 0.57% dibutyltin maleate (HydroPolymers Ltd., County Durham, UK).

### 3.5. Methylene Blue Number (MBN) and Iodine Number (IN) Determination

The methylene blue (MB) number is defined as the maximum amount of dye adsorbed by 1 g of adsorbent. In this assay, 10 mg of activated carbon is placed in contact with 10 mL of an MB solution at different concentrations (10, 25, 50, 100, 250, 500 and 1000 mg/L) for 24 h at room temperature. The initial and remaining concentrations of MB were analyzed using a UV/Vis spectrophotometer (SP-2000, Tbtscietech, Nanjing, China) at 645 nm.

The amount of MB adsorbed from each solution is calculated by Equation (7).

(7) $$q_{eq}=\frac{\left(C_{0}-C_{e}\right)\times V}{M}$$

where *C*_0_ (mg/L) is the initial concentration of the MB, while *C_e_* (mg/L) is the concentration of the MB solution at equilibrium time, *V* (L) is the volume of the MB solution and *M* (g) is the mass of the adsorbent.

The iodine number is determined according to the ASTM D4607-94 method. The iodine number is defined as the milligrams of iodine adsorbed by 1 g of carbon when the iodine concentration of the filtrate is 0.02 N [68].

### 3.6. Boehm Titration

Surface functionality of AC samples was assessed via a modified Boehm titration procedure [68]. The supernatants were then back-titrated with NaOH or HCl to determine the surface functional groups, with each analysis performed in triplicate. The total number of acidic groups (carboxylic, phenolic and lactone) was determined by neutralization with NaOH, while the number of basic sites was calculated from the amount of HCl required for their neutralization. The quantities of surface functional groups were expressed in milliequivalents (meq) per gram of sample.

### 3.7. Assessment of Adsorption of Uremic Toxin Precursors Using Porous Carbons

For each PC sample, 20 mg was weighed in triplicate for each incubation time (total of 9 replicates), placed into Eppendorf tubes and pre-equilibrated with 4 mL of phosphate-buffered saline (PBS) in a shaking incubator at 37 °C for 4 h. The PCs were then centrifuged at 8000 rpm for 3 min, and the supernatant was removed. Fresh PBS solutions, each supplemented with p-cresol, indole and IAA at 5 mM (10 mL total), were incubated in a shaking incubator at 37 °C for 1 h. After incubation, 3.75 mL of the spiked PBS solution (simulating the pH and ionic strength of gastrointestinal fluid) was added to each PC sample and control. Samples were further incubated at 37 °C with shaking and collected at time points of 15, 30, 45, 60, 90, 120, 150 and 180 min. At each time point, the PCs were centrifuged at 8000 rpm for 3 min, and the supernatant (n = 3 per time point) was transferred to Eppendorf tubes and stored at −80 °C before the analysis of the remaining concentrations using UV-Vis spectroscopy technique. First, absorption maximum peaks were assessed at 277 nm for p-cresol, 274 nm for indole and 520 nm for IAA, at which the measurements of the absorption intensity (absorbance) were later determined. Secondly, calibration curves of “concentration vs. absorbance” were built in order to assess the remaining PBUT precursors concentrations within the range from 0 to 250 µmol/L.

Properly diluted final solutions concentrations of the PBUT precursors in the supernatant were determined by an UV–Vis scanning spectrophotometer (Jenway 6705, Cole-Parmer, UK), using the calibration curves obtained within the range from 0 to 250 µmol/L, which are shown in Figure S4. All experiments were performed in triplicate, and the results were presented as the average. The adsorption capacity (*q_t_*) at a time point (*t*) in mmol/g and removal efficiency (*RE*, %) of adsorbate were calculated according to Equations (8) and (9) as follows:

(8) $$q_{t}=\;\frac{\left(C_{0}-\;C_{t}\right)\;*\;V}{W}$$

(9) $$RE=\frac{\left(C_{0}-C\right)}{C_{0}}\;*\;100\%$$

where *C*_0_ and *C_t_* (mmol/L) are the initial and final concentrations at respective time points (*t*) of an adsorbate solution. *V* (L) is the volume of the solution (mL), and *W* (g) is the weight of an adsorbent (0.02 g). The experimental results of contact time and initial adsorbate concentration were used for adsorption kinetics and isotherm study, respectively (Table 8, Figure 15 and Figure 16).

## 4. Conclusions

This study reported successful development and characterized a series of porous carbon (PC) adsorbents from rice husk (RH) to investigate the removal of phenolic and heterocyclic metabolites (p-cresol, indole and indole-3-acetic acid (IAA)). The findings demonstrate that the sorption efficiency is defined by a complex interaction between textural properties and surface chemistry.

The primary factor controlling the adsorption capacity is the micropore design. The direct correlation between the sorption performance series (PC-N1 > PC-N2 > PC-P) and their respective micropore volumes (V_DR_) highlights the critical role of micropores in the uptake of these molecules. This was further confirmed by pore size distribution analysis, which revealed that samples with characteristic peaks in the ultramicropore region (<1 nm) exhibited superior sorption.

Surface functionalization, achieved through different activation methods, also played a crucial role. While PC-N1 and PC-N2 showed comparable high sorption capacities for p-cresol and indole, their different surface chemistries and pHpzc values revealed the specific adsorption mechanisms. The basic nature of PC-N2 (pHpzc = 8.78) enhanced its efficiency for anionic p-cresol and polar indole via Lewis acid–base and π–π interactions. In contrast, the phosphate groups on the PC-P surface likely created repulsive interactions with anionic analytes, explaining its comparatively lower removal values even while having a large specific surface area.

In summary, this research validates that various activation protocols can effectively tune the pore structure and surface chemistry of RH-derived carbons. The resulting materials are highly promising, non-toxic candidates for use as oral adsorbents or in filtration systems for the targeted removal of specific polar micropollutants from biological and aquatic environments.

The toxicity of the PCs was tested in vitro using LDH and MTS assays with no evidence of cytotoxicity. The results suggest that these PCs may be used as oral adsorbents (enterosorbents), as well as components of filters for polar micropollutant removal from aqueous media.

## Figures and Tables

**Table 1:** Specific surface area (S), pore volume (V), methylene blue number (MBN) and iodine number (IN) of PC samples calculated using different methods.

Sample Code	S_BET_, m^2^/g	S_QSDFT_, m^2^/g	V_BJH_, cm^3^/g	V_DR_, cm^3^/g	V_QSDFT_, cm^3^/g	V_DR_/ V_QSDFT_	D_QSDFT_, nm	MBN, mg/g	IN, mg/g	IN/ MBN
PC-N1	2690	2330	0.85	1.01	1.84	0.549	0.89	1571	2810	1.79
PC-N2	1410	1360	0.39	0.63	0.91	0.692	0.82	621	1545	2.48
PC-P	1700	1400	1.15	0.48	1.56	0.308	1.10	538	960	1.78

**Table 2:** Chemical composition and relative atomic ratio determined by EDS and XPS analyses of PC samples.

Sample Code	PC-N1	PC-N2	PC-P
Semi-quantitative element content of PC samples by EDS microanalysis (at. %)			
C	92.4 ± 0.3	96.1 ± 0.3	92.4 ± 0.3
O	6.7 ± 0.3	3.0 ± 0.3	3.5 ± 0.3
Si	0.1	0.1	0.1
P	-	-	2.5
S	0.1	0.1	0.1
Na	-	-	1.3
K	0.7	0.8	-
Chemical composition of PCs and relative atomic ratio determined by XPS (at. %)			
C	90.9 ± 0.8	96.8 ± 0.2	94.4 ± 0.3
O	6.7 ± 0.5	1.4 ± 0.1	5.1 ± 0.3
N	1.3	1.4 ± 0.2	-
Si	1.1 ± 0.2	0.4	0.06
P	-	-	0.49 ± 0.1

**Table 3:** High-resolution XPS Narrow Scan Results for PC-N1 Sample.

Surface Functional Groups	Peak BE, eV	Atomic % (Average)	Std. Dev.
Carbon-containing functional groups			
Hydrocarbon (C–C, C–H)	284.94	85.56	0.2
Amine (C–N)	285.93	7.10	0.5
Alcohol/Ether (C–O)	286.64	7.35	0.4
Nitrogen-containing functional groups			
Amine (–C–NH_2_)	398.09	21.84	0.2
Imine (–C=NH)	399.34	53.12	1.2
Nitro (–C–NO_2_)	400.59	17.08	0.4
Aminiumion (–C–NH_3_^+^)	402.13	7.96	0.4
Oxygen-containing functional groups			
Nitro (–NO_2_) pyridine-N-oxide	530.39	21.29	1.2
Carbonyl (–C=O)	531.6	35.65	1.0
Carboxylic (–O–C=O)	532.6	36.8	0.1
Phenolic (–C–OH)	533.3	6.2	0.3

**Table 4:** High-resolution XPS Narrow Scan Results for PC-N2 Sample.

Surface Functional Groups	Peak BE, eV	Atomic % (Average)	Std. Dev.
Carbon-containing functional groups			
Hydrocarbon (C–C, C–H)	284.56	81.0	0.3
Amine (C–N)	285.34	12.2	0.3
Alcohol, Ether (C–O)	286.26	6.8	0.1
Nitrogen-containing functional groups			
Pyridinic (N–6)	398.36	55.0	3.2
Pyrrolic/Pyridone (N–5)	400.09	29.5	1.4
Quaternary (N–Q)	401.55	15.6	1.9
Oxygen-containing-functional groups			
Carbonyl (–C=O)	530.5	14.4	4.8
C–OH phenol groups	532.02	26.4	3.6
C–O–C ether groups	533.31	52.1	6.6
Water	535.1	7.1	1.9

**Table 5:** High-resolution XPS scanning results: series for P2p spectra of PC-P sample.

Surface Functional Groups	Peak BE, eV	Atomic % (Average)	Std. Dev.
Carbon-containing functional groups			
Hydrocarbon (C–C, C–H) Graphitic carbon	284.63	88.5	0.2
Alcohol, ether groups and/or C–O–P	286.07	5.2	0.3
Carbon in carbonyl groups	286.91	4.4	0.5
Carboxyl and/or ester groups	288.08	1.9	0.8
Phosphorus-containing functional groups			
C–O–PO_3_ groups in (pyro)phosphates (PO_4_ tetrahedra)	133.04	66.2	0.3
Phosphorus bound to oxygen in (P–O–P)	134.42	33.8	0.7
Oxygen-containing-functional groups			
=O in carbonyl, carboxyl and phosphates (C=O and P=O)	531.01	13.5	0.4
Singly bonded oxygen (–O–) in C–O and C–O–P groups	532.84	60.6	0.3
C–O–C ether groups	534.18	25.9	0.2

**Table 6:** Results of pseudo-first- and pseudo-second-order kinetic modeling for PBUT precursor adsorption.

Sample	Adsorbate	q_e_cal, mmol/g	*k*_1_, min^−1^	R^2^	q_e_cal, mmol/g	*k*_2_, min^−1^	R^2^
		Pseudo-First Order			Pseudo-Second Order		
PC-N1	Indole	0.060738	0.03510	0.46525	2.42	18.6	0.999
	P-cresol	0.206518	0.0699	0.79073	2.43	9.86	0.999
	IAA	0.9663	0.09676	0.79186	2.01	2.21	0.999
PC-N2	Indole	0.18233	0.03897	0.76606	2.19	5.39	0.999
	P-cresol	0.450391	0.06824	0.80238	2.24	1.71	0.992
	IAA	1.04	0.06780	0.71673	1.49	0.33	0.998
PC-P	Indole	1.346461	0.05875	0.88319	1.74	0.91	0.997
	P-cresol	0.370582	0.01423	0.98828	1.60	0.77	0.999
	IAA	0.48	0.07350	0.89949	0.88	0.09	0.999

**Table 7:** Comparative data for carbon-based adsorbents used for p-cresol, indole and IAA removal.

	Adsorbent	S_BET_, m^2^/g	V_tot_, cm^3^/g	V_micro_, cm^3^/g	V_meso_, cm^3^/g	C_0_, mg/L	q_max_, mmol/g	Ref.
p-cresol	Methanesulfonic-acid-activated carbon	-	-	-	-	400	0.81	[50]
	NaOH-activated carbon coconut shell	520	0.31	0.16	0.15	1000	2.37	[13]
	AC/cellulose acetate	375	0.35	0.021	-	50	0.95	[51]
	Parthenium-based activated carbon	260	-	-	-	1000	0.56	[52]
	K_2_CO_3_-activated pine wood carbon	21	0.002	-	-	100	0.05	[53]
	Coconut-shell-based activated char					500	0.31	[54]
	KOH-activated corn silk carbon	1363	0.375	0.491	0.642	500	4.4	[50]
	CO_2_-activated corn silk carbon	934	0.166	0.260	0.495	500	3.7	[55]
	PC-N1	2690	1.84	1.01	0.85	541	2.43	This work
	PC-N2	1410	0.91	0.63	0.39	541	2.24	
	PC-P	1700	1.56	0.48	1.15	541	1.6	
indole	Resorcinol-derived mesoporous carbon	795	-	-	-	100	2.72	[56]
	Ketjen carbon black	1422	-	-	-	100	3.8	[50]
	NDPC-500	909	0.43	0.33	0.10	1500	5.01	[57]
	NDPC-600	1293	0.60	0.47	0.13	1500	5.95	[57]
	NDPC-700	1944	0.92	0.71	0.21	1500	7.35	[57]
	NDPC-800	2166	0.94	0.87	0.07	1500	6.36	[57]
	AC	1016	0.57	-	-	300	0.54	[58]
	CuCl/AC	897	0.51	-	-	300	1.43	[58]
	H_3_PO_4_-activated wood-based carbon	2330	-	1.36	-	1000	1.08	[59]
	AST-120	2384	1.2	0.86	0.3	100	1.7	[60]
	KOH-RF-F127(3)	1563	0.78	0.29	0.48	100	2.56	[60]
	KOH-RF-F127(2)	1219	0.66	0.26	0.38	100	2.3	[60]
	KOH-RF-F127 (1)	1328	0.52	0.21	0.29	100	1.85	[60]
	RF-F127	423	0.35	0.07	0.27	100	0.23	[60]
	Granular AC	355	0.19		0.029	1000	0.32	[60]
	PC-N1	2690	1.84	1.01	0.85	586	2.42	This work
	PC-N2	1410	0.91	0.63	0.39	586	2.19	
	PC-P	1700	1.56	0.48	1.15	586	1.74	
IAA	NPCA	328	0.21	0.12	-	5	1.19	[61]
	Carbotech AC	525	-	0.24	0.04	500	1.79	[62]
	Starbon A300	100	-	0.03	0.42	500	0.98	[62]
	Starbon A500	409	-	0.15	0.46	500	1.28	[62]
	Starbon A800	459	-	0.17	0.33	500	1.34	[62]
	PC-N1	2690	1.84	1.01	0.85	876	1.97	This work
	PC-N2	1410	0.91	0.63	0.39	876	1.44	
	PC-P	1700	1.56	0.48	1.15	876	0.85	

**Table 8:** The relationship between adsorption capacity and surface chemistry of phenolic and indolic molecules and PCs.

Physicochemical Characteristics of Phenolic and Indolic Molecules									
	P-cresol			Indole			Indole-3-acetic acid		
	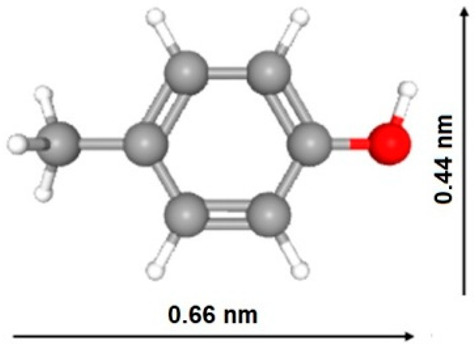 CH_3_C_6_H_4_OH			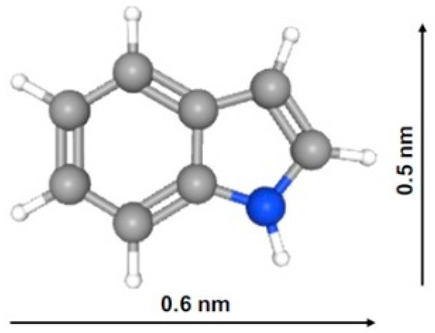 C_8_H_7_N			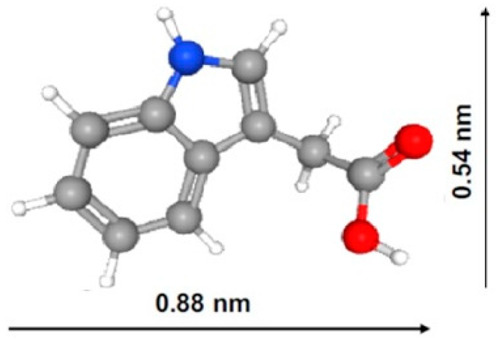 C_8_H_8_NCOOH		
Cross-sectional area, nm^2^	~0.29			~0.3			~0.48		
Molar mass, g/mol	108.14			117.15			175.18		
Solubility in water, g/L	21.5			3.56			1.5		
Solubility in water, mmol/L	198.8			30.29			8.56		
p*K_a_*	10.3			p*K_b_*~17.6			4.54		
Time	Removal efficiency, RE ([(*C*_0_ − *C*_t_)/*C*_0_] × 100%)								
(min.)	PC-N1	PC-N2	PC-P	PC-N1	PC-N2	PC-P	PC-N1	PC-N2	PC-P
15	80.24	41.45	52.56	87.28	73.19	39.52	61.18	39.89	23.35
30	91.34	81.89	54.89	94.57	83.97	65.24	67.51	42.42	26.48
45	92.45	84.06	55.34	94.90	84.77	65.38	69.91	45.12	28.18
60	93.66	86.32	56.11	95.18	85.7	65.84	72.41	48.02	29.86
90	93.71	86.89	57.24	95.47	86.13	67.07	73.27	49.66	30.95
120	93.75	87.36	58.01	95.75	86.68	68.6	74.29	51.61	31.93
150	94.44	88.02	60.13	95.91	86.76	68.68	74.63	53.26	32.18
180	95.14	88.64	62.18	96.05	86.9	68.7	74.93	54.83	32.28
Adsorption and surface structural characteristics of PCs vs. phenolic and indolic adsorbates									
RE_15_/RE_180_, %	84.34	46.76	84.53	90.87	84.22	57.53	81.65	72.75	72.34
q_t=180_, mmol/g	2.43	2.24	1.60	2.42	2.19	1.74	1.97	1.44	0.85
q_t_/V_DFT_	1.32	2.46	1.03	1.32	2.40	1.12	1.07	1.6	0.54
q_t_/V_DR_, mmol/cm^3^	2.41	3.56	3.33	2.40	3.48	3.63	1.95	2.29	1.77
IN/MBN	1.79	2.48	1.78	1.79	2.48	1.78	1.79	2.48	1.78
V_DFT_, m^3^/g	1.84	0.91	1.56	1.84	0.91	1.56	1.84	0.91	1.56
V_DR_, cm^3^/g	1.01	0.63	0.48	1.01	0.63	0.48	1.01	0.63	0.48
V_DR_/V_QSDFT_	0.549	0.692	0.308	0.549	0.692	0.308	0.549	0.692	0.308
PC pH_pzc_	5.71	8.78	6.14	5.71	8.78	6.14	5.71	8.78	6.14
Boehm acidic groups, e.g., –C–O–PO_3_, –C–OO^−^, Ph–O^−^, meq/g	1.36	0.90	3.83	1.36	0.90	3.83	1.36	0.90	3.83
Boehm basic groups, e.g., –C–NH_3_^+^, amine, pyridinic, pyrrolic, quaternary, meq/g	1.90	3.40	-	1.90	3.40	-	1.90	3.40	-

## Data Availability

The original contributions presented in this study are included in the article/Supplementary Material. Further inquiries can be directed to the corresponding authors.

## Supplementary Materials

The following supporting information can be downloaded at: https://www.mdpi.com/article/10.3390/ijms27020804/s1.
