# Supplementary material for: Assessment of Phenolic and Indolic Compounds Removal from Aqueous Media Using Lignocellulose-Derived Surface-Modified Nanoporous Carbon Adsorbents: A Comparative Study
Source: Int J Mol Sci. 2026 Jan 13;27(2):804. doi: 10.3390/ijms27020804 (PMC12840695; doi:10.3390/ijms27020804)
Supplement: Supplementary file 1 [file ijms-27-00804-s001.zip › ijms-4010840-supplementary-update Figure S4.pdf]

Assessment of Phenolic and Indolic Compounds Removal from Aqueous Media using Lignocellulose-derived Surface Modified Nanoporous Carbon Adsorbents: A Comparative Study

Jakpar Jandosov [1,2,*], Dmitriy Chenchik [1], AlzhanBaimenov [1,3,4,*], Joaquin Silvestre-Albero [5], Maria Bernardo [6], Seitkhan Azat [3], Yerlan Doszhanov [1,2], Aitugan Sabitov [1,2], Rosa Busquets [7], Carol Howell [8], Sergey Mikhalovsky [9,10] and Zulkhair Mansurov [1,2]

[1] Institute of Combustion Problems, 172, Bogenbay Batyr St., Almaty 050000, Kazakhstan; dmitriychenchik@gmail.com (D.C.); doszhanov_yerlan@mail.ru (Y.D.); aitugans@mail.ru (A.S.); zmansurov@kaznu.kz (Z.M.)

[2] Faculty of chemistry and chemical technology, Al-Farabi Kazakh National University, 71, Al-Farabi Avenue, Almaty 050012, Kazakhstan

[3] Laboratory of Engineering Profile, Satbayev University, 122/22, Baitursynov St., Almaty 050012, Kazakhstan; seytkhan.azat@gmail.com

[4] Laboratory of EPR spectroscopy, Institute of Physics and Technology, 11, Ibragimov St., Almaty 050000, Kazakhstan

[5] Laboratorio de Materiales Avanzados, Departamento de Química Inorgánica, Universidad de Alicante, 03690 Alicante, Spain; joaquin.silvestre@ua.es

[6] Departamento de Química (DQ), Faculdade de Ciências e Tecnologia (FCT), Universidade Nova de Lisboa (UNL), 2829-516 Caparica, Portugal; maria.b@fct.unl.pt

[7] School of Life Sciences, Pharmacy and Chemistry, Kingston University, Penrhyn Road, Kingston upon Thames KT1 2EE, UK; r.busquets@kingston.ac.uk

[8] Enteromed Ltd., 85 Great Portland St, London W1W 7LT, UK; howellcarol32@gmail.com

[9] ANAMAD Ltd., Sussex Innovation Centre, Science Park Square, Falmer, Brighton BN1 9SB, UK; sergeymikhalovsky@gmail.com

[10] Chuiko Institute of Surface Chemistry, 17, General Naumov St., Kyiv 03164, Ukraine

* Correspondence: jandosovj@gmail.com (J.J.); alzhan.baimenov@satbayev.university (A.B.)

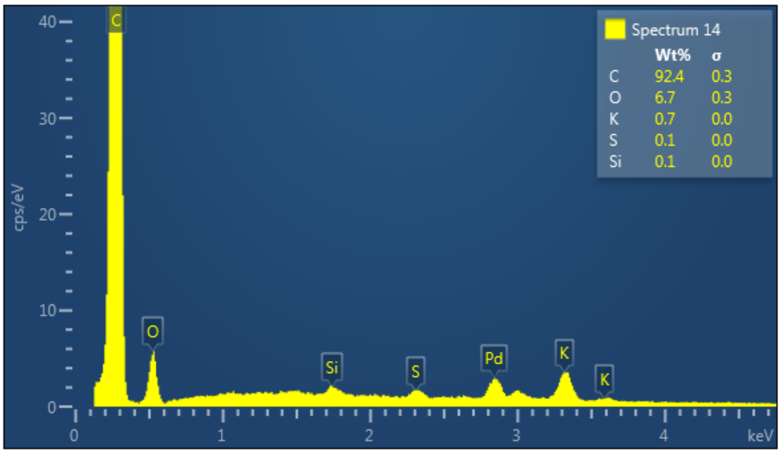

(a)

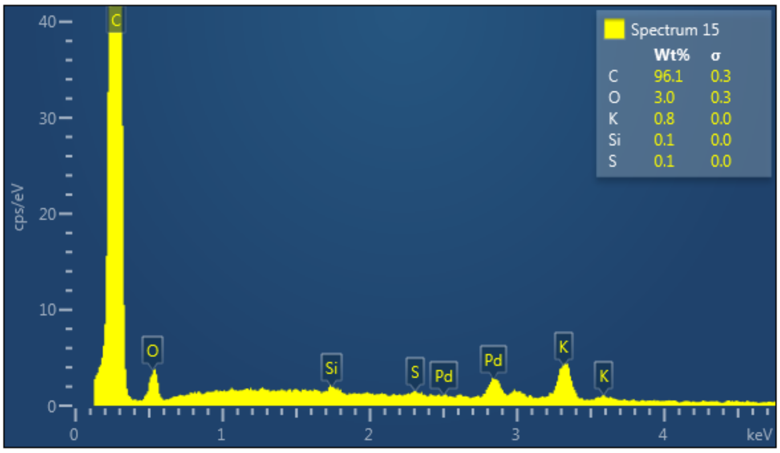

(b)

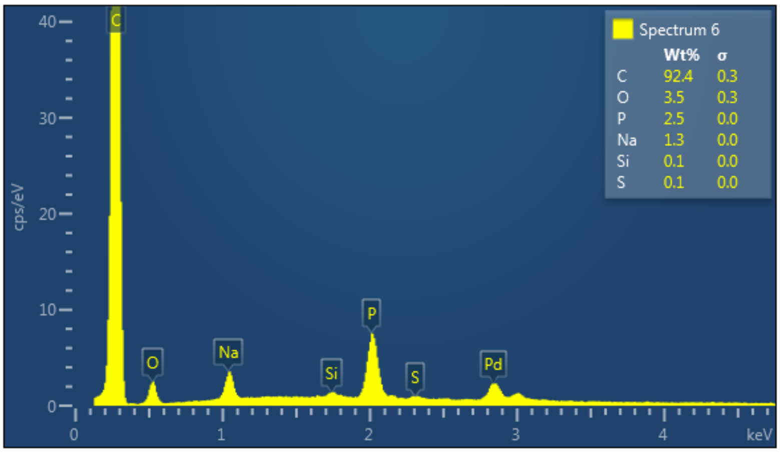

(c)

Figure S1. EDS energy spectra of different PC samples: (a) PC-N1; (b) PC-N2; (c) PC-P.

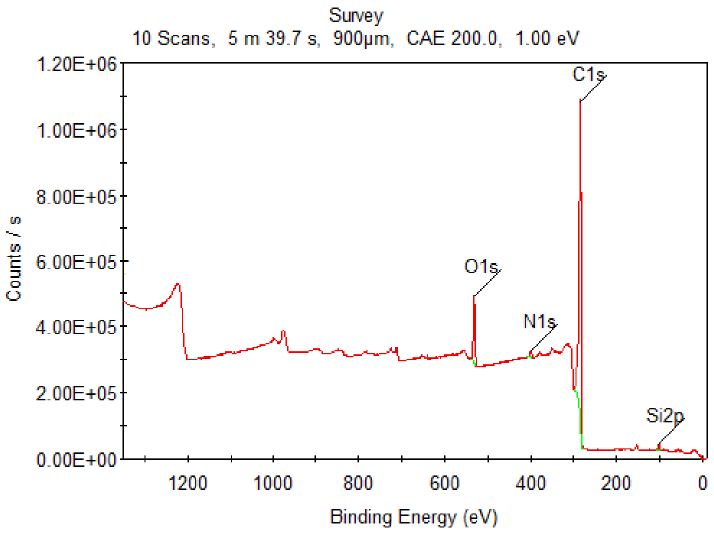

(a)

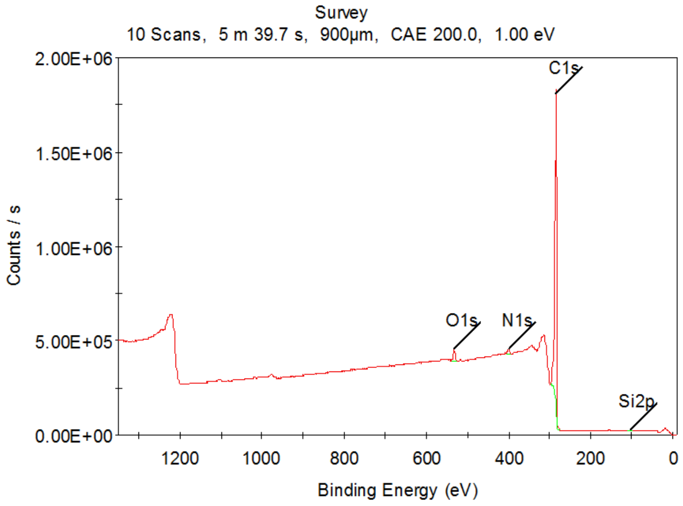

(b)

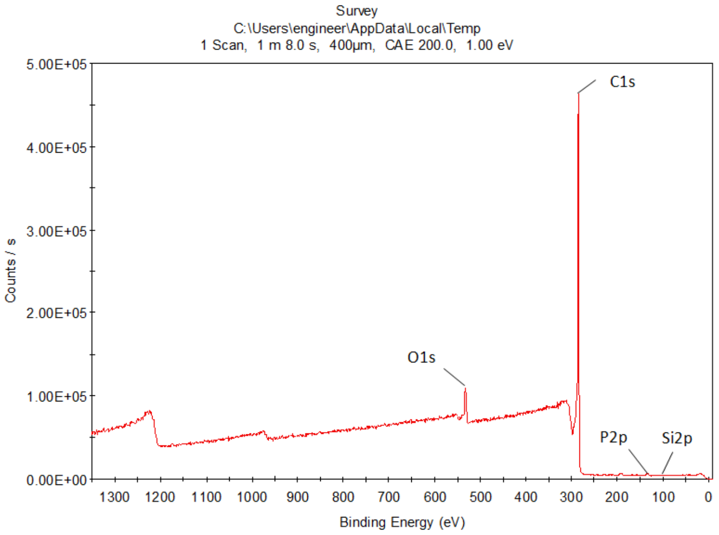

(c)

Figure S2. XPS full spectrum of different PC samples: (a) PC-N1; (b) PC-N2; (c) PC-P.

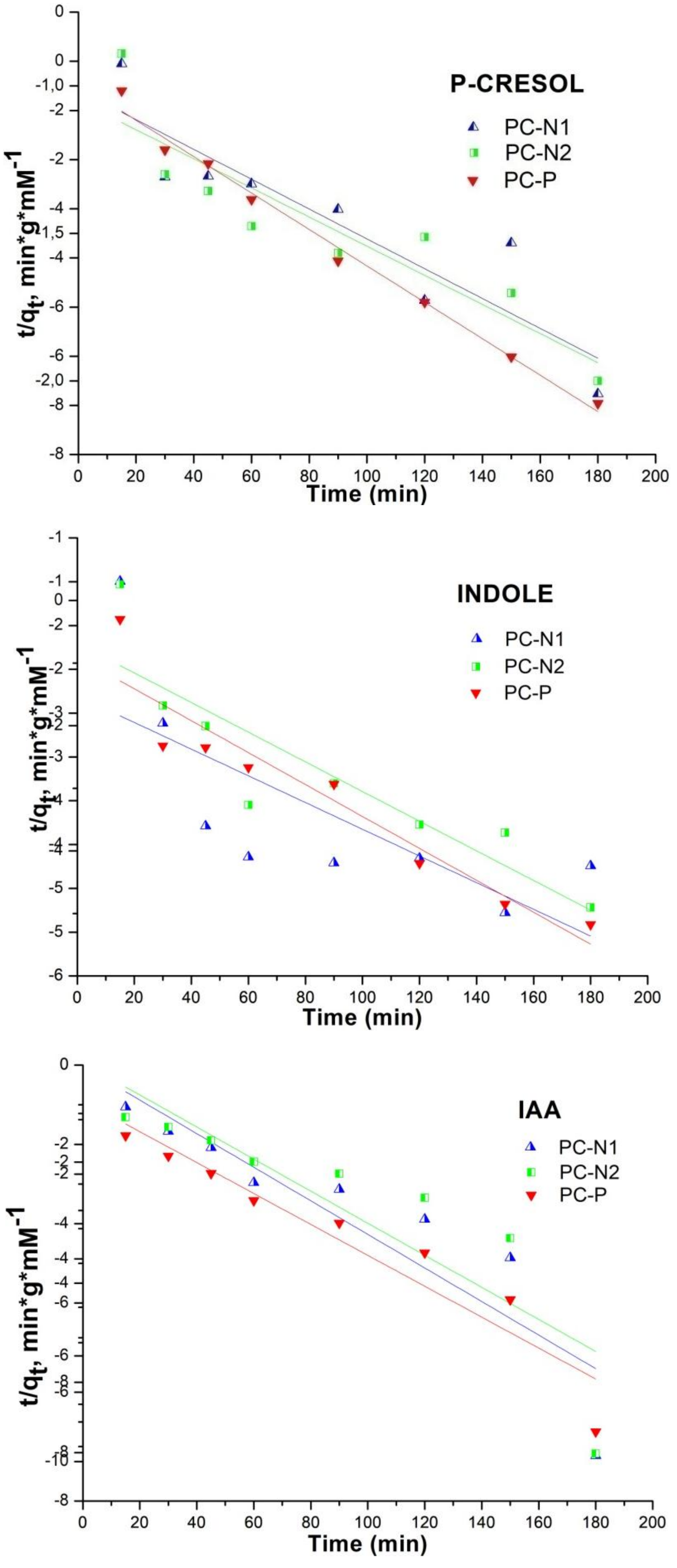

Figure S3. Linearized pseudo-first order kinetic models for p-cresol, indole and IAA, adsorbed by PC-N1, PC-N2 and PC-P samples.

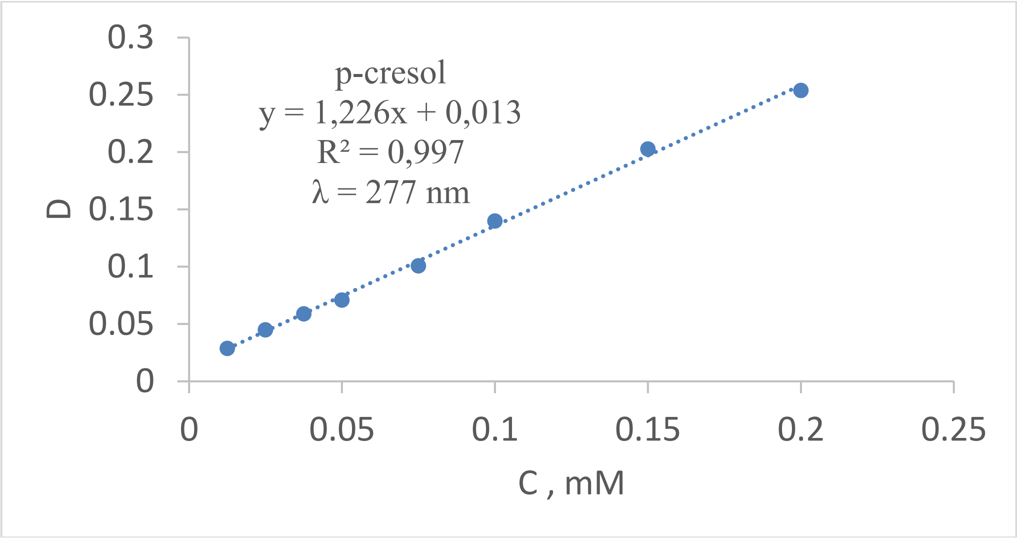

(a)

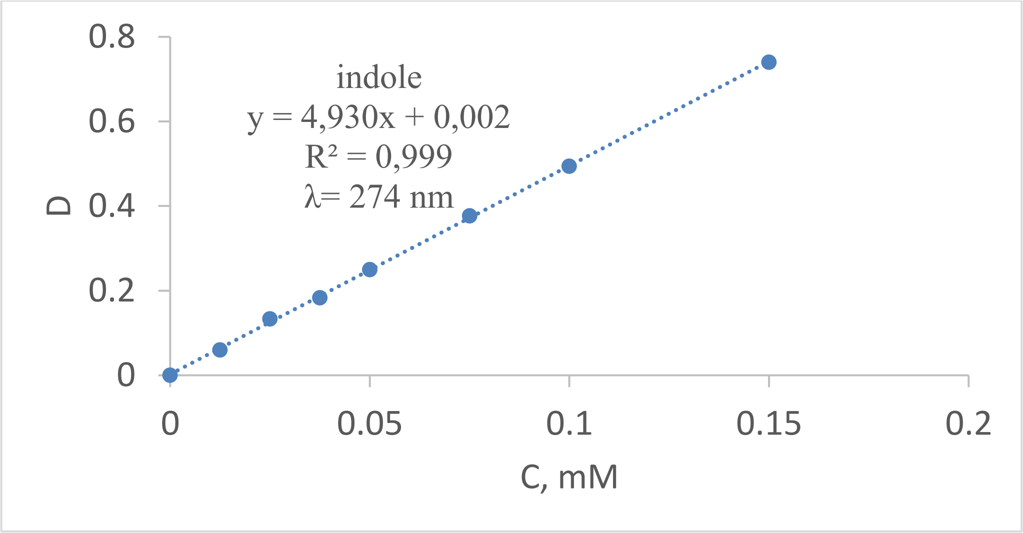

(b)

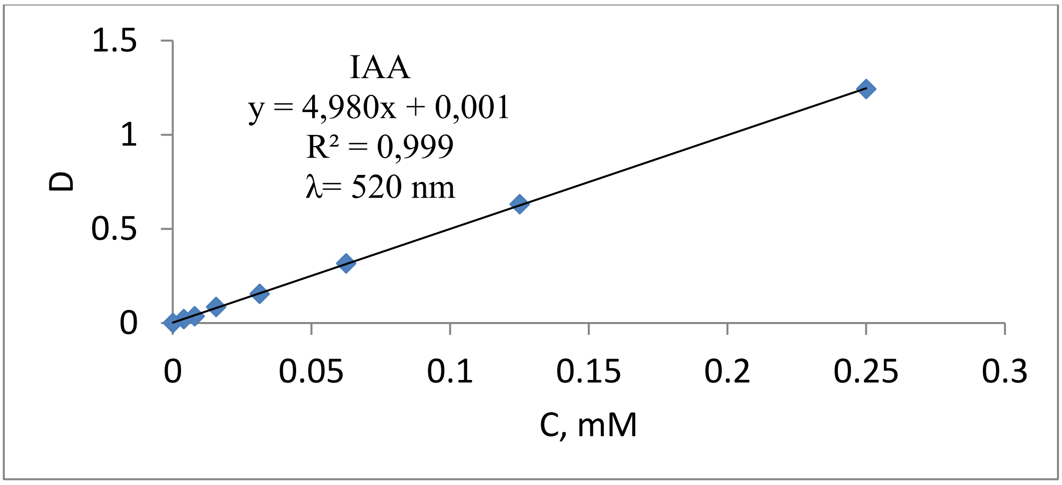

(c)

Figure S4. Linearized UV-vis calibration curves of absorption D vs. concentration C (mM) for p-cresol (a), indole (b) and IAA (c).
